# Inducing hardening and healability in poly(ethylene-*co*-acrylic acid) *via* blending with complementary low molecular weight additives†

**DOI:** 10.1039/c8ra09597c

**Published:** 2018-12-12

**Authors:** Benjamin C. Baker, I. German, Gary C. Stevens, Howard M. Colquhoun, Wayne Hayes

**Affiliations:** Department of Chemistry, University of Reading Whiteknights, Reading RG6 6AD UK w.c.hayes@reading.ac.uk +44 (0)118 378 6331 +44 (0)118 378 6491; Gnosys Global Ltd. 17-18 Frederick Sanger Road, The Surrey Research Park, Guildford Surrey GU2 7YD UK

## Abstract

The design and synthesis of low molecular weight additives based on self-assembling nitroarylurea units, and their compatibility with poly(ethylene-*co*-acrylic acid) copolymers are reported. The self-assembly properties of the low molecular weight additives have been demonstrated in a series of gelation studies. Upon blending at low percentage weights (≤5%) with poly(ethylene-*co*-acrylic acid) the additives were capable of increasing the stress and strain to failure when compared to the parent copolymer. By varying the percentage weight of the additive as well as the type of additive the mechanical properties of poly(ethylene-*co*-acrylic acid) could be tailored. Finally, the healability characteristics of the blends were improved when compared to the original polymer *via* the introduction of a supramolecular ‘network within a network’.

## Abstract 2

Blending nitroarylurea gelators with poly(ethylene-*co*-acrylic acid) copolymers improves the mechanical and healing properties of the bulk polymer *via* ‘network within a network’ formation.

## Graphical abstract

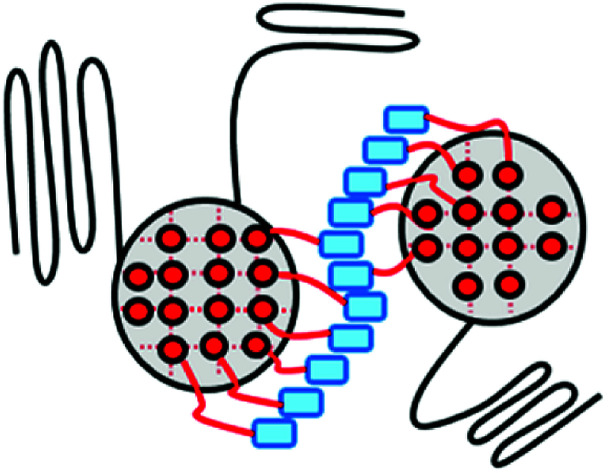

## Design, system, application

In the light of limited petroleum feedstocks, the enhancement of the mechanical properties of known polymeric materials and improvement of polymer-based product lifetimes are key targets in polymer science. This study outlines a systematic approach to these goals whereby blends are created between commercially available polymers and small quantities of functionalised low molecular weight additives, specifically additives which are capable of forming stable gel networks. The bulk polymers used in this study associate into networks *via* non-covalent interactions and are healable because of their self-assembling character. The molecular additives were designed to bind *via* non-covalent interactions with the bulk polymer and also to self-associate in order to create an alternative network to that found in the bulk polymer alone. Creating a ‘network within a network’ has been shown in this study to improve both the mechanical characteristics and healing ability of the bulk polymer. This approach to the enhancement of properties of existing polymer systems *via* blending with complementary low molecular weight additives thus offers an alternative route to the development of healable polymeric-based products without the need for the synthesis of complex polymer structures.

## Introduction

Ethylene-based polymers have found widespread use as protective coatings.^1^ The toughness of phases arising from polyethylene sequences and the ability to manipulate mechanical properties of the co-polymer, *via* variations in comonomer structure and relative content, allow access to materials suitable for a wide range of applications.^2^ However, when damaged, repair for many of these ethylene-based copolymers cannot be realised and thus the system fails as a protection mechanism (such as cracking within electrical cabling). The introduction of healability within a polymeric protection system *via* the use of copolymer functionalities capable of non-covalent associations has been demonstrated successfully by Kalista *et al.*^3,4^

Several distinct approaches to the realisation of polymer-based healable systems have been reported in the literature.^5–7^ The “encapsulation” and “reversible/irreversible covalent bond” approaches offer alternative routes to healability within polymer matrices.^8^ However, these approaches have distinct practical limitations including a limited number of break-heal cycles (encapsulation and irreversible covalent bond approaches), reduced toughness of the repaired system and the need to implement external stimuli to initiate the healing process (reversible/irreversible covalent bond approach).^9^ An alternative approach whereby non-covalent sacrificial bonds, for example hydrogen bonds, are placed throughout polymeric networks have allowed access to healability^10^*via* bond dissociation and re-association.^11^ However, the inevitable compromise between the thermal and kinetic stimuli required to initiate healing and the ability of the system to offer viable protection must be considered.^12,13^

The self-healing mechanism of ethylene carboxylic acid copolymers, such as poly(ethylene-*co*-methacrylic acid), has been modelled as a two-stage system relying upon a supramolecular rearrangement and melting of the polymeric crystalline phase.^3,4^ In this model sacrificial supramolecular bonds (intermolecular hydrogen bonding between carboxylic acid moieties) are able to dissociate at lower temperatures and enable healability. At higher temperatures melting of the crystalline polyolefin domains enables copolymer flow and processability. This model has been extended to the random copolymer poly(ethylene-*co*-acrylic acid).^3,4,13^

**Fig. 1:**
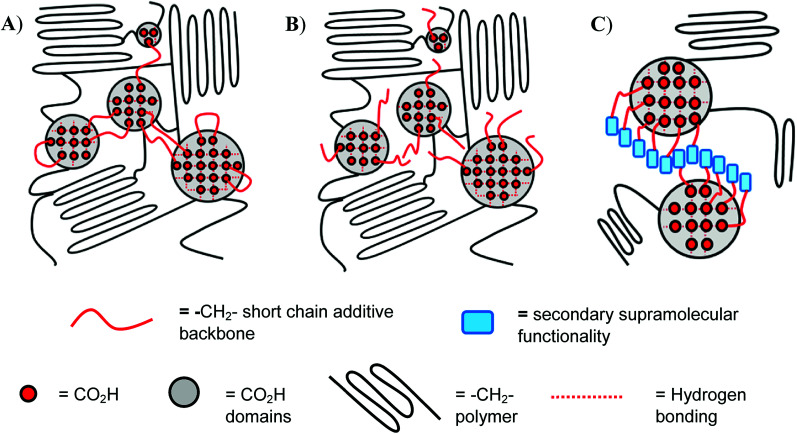
Insertion of; (A) dicarboxylic acid additive (sebacic acid), (B) mono-carboxylic acid additive (dodecanoic acid), (C) creation of ‘network within a network’ *via* functionalised carboxylic acids additives to enable secondary supramolecular interactions into poly(ethylene-*co*-acrylic acid).

In the present paper we report the introduction of low molecular weight additives based upon hydrogelators^14–16^ featuring the nitroarylurea receptor motif into a range of ethylene-carboxylic acid copolymers as a means of enhancing both the mechanical properties and healability characteristics of the bulk phase.^17–23^ It is known that creation of a secondary ‘soft’ network within a well-defined polymer network, by introduction of small-molecule, gel-forming additives into copolymers,^22–24^ promotes healing at lower temperatures, provided that such additives interact with the moieties within the bulk polymer that are responsible for healing (in the present work the carboxylic acid groups, see Fig. 1). The small-molecule additives also strengthen the polymer matrix at lower temperatures (*ca*. 20 °C) *via* increased ordering within the bulk (at temperatures < *T*_g_) in agreement with studies on precisely-defined poly(ethylene-*co*-acrylic acid) copolymers reported by Middleton *et al.*^25^

## Results and discussion

### Low molecular weight additive synthesis and characterisation

**Fig. 2:**
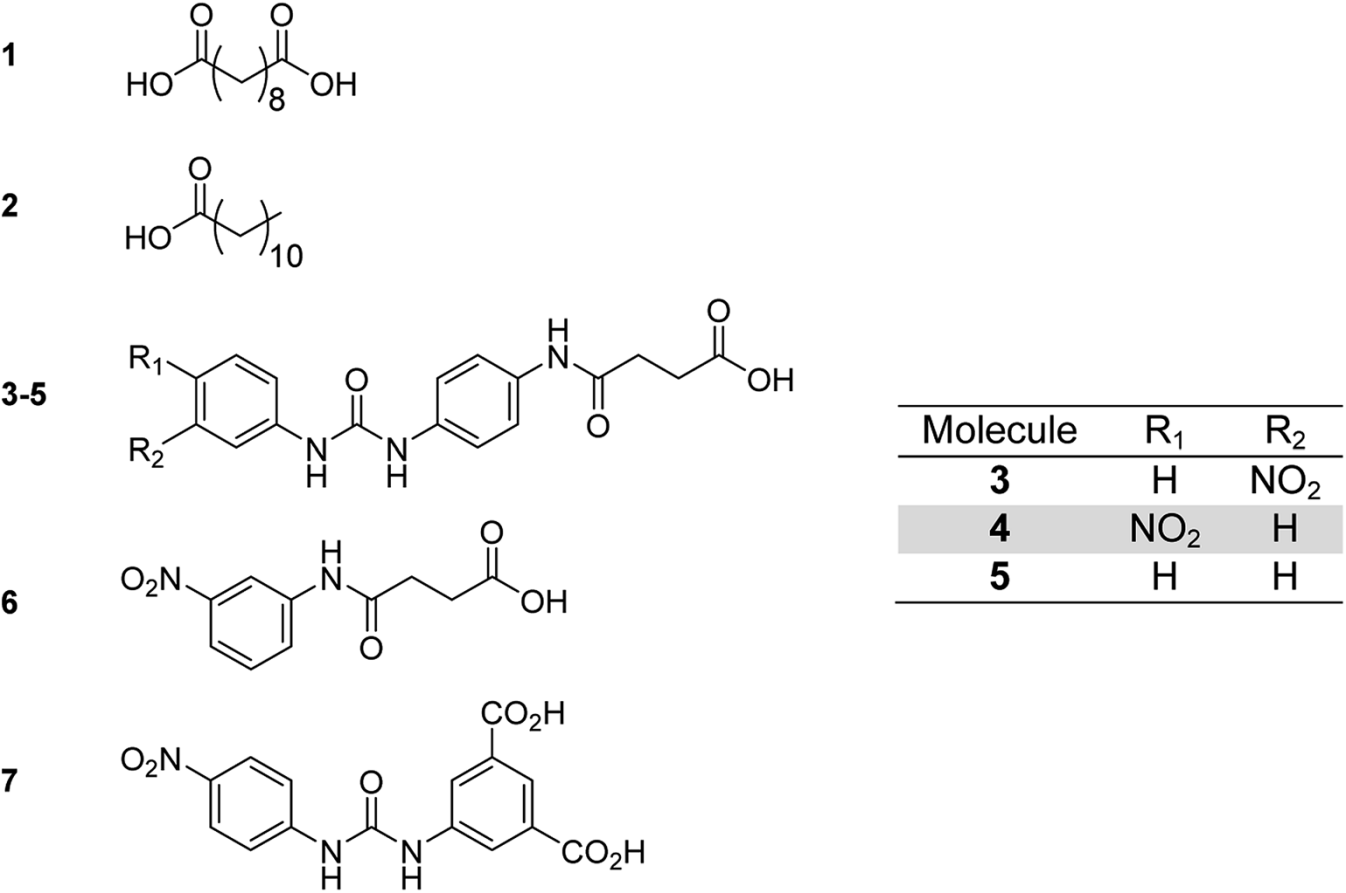
Low molecular weight additives showing: sebacic acid 1, dodecanoic acid 2, functionalised carboxylic acids 3–7.

Each of the additive molecules 1–6 (Fig. 2) was designed to interact with the carboxylic acid hydrogen bonding domains present in poly(ethylene-*co*-acrylic acid).

**Scheme 1:**
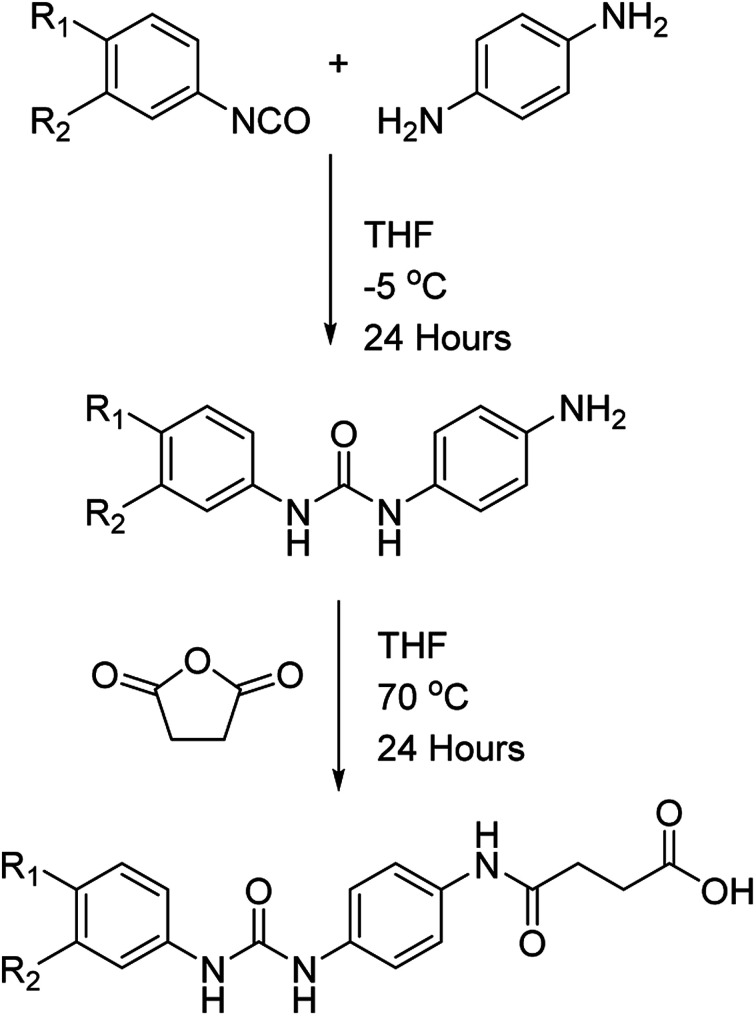
Generic synthesis of gelator molecules 3–5.

The dicarboxylic acid, sebacic acid (1), and the mono acid, dodecanoic acid (2), were used as received. Carboxylic acids 3–5 were synthesised *via* a two-step process (Scheme 1), with each respective amine-functionalised diaryl urea being formed from the corresponding aryl isocyanate and *p*-phenylenediamine.^10*d*,26^ The carboxylic acids were then generated *via* a ring opening reaction of the remaining amino group with succinic anhydride.

The successful synthesis of each carboxylic acid was confirmed by IR, ^1^H and ^13^C NMR spectroscopic analysis in addition to mass spectrometry (see the ESI, Fig. S1–S9†). For example, ^1^H NMR analysis of the carboxylic acid 3 showed that the primary amine resonances present in the starting material were replaced by an amide-NH resonance at 9.83 ppm (see Fig. S1 in the ESI†). These spectroscopic data were in agreement with the ^13^C NMR analysis which revealed the presence of three different carbonyl resonances (*e.g.* 175.8, 169.7 and 153.8 ppm) corresponding to the urea, amide and carboxylic acid groups present in the product (Fig. S1†). Further proof of the successful synthesis of 3 was evident from the IR spectrum (Fig. S3†) which exhibited three distinct absorption bands (at 1696, 1671 and 1655 cm^−1^, respectively) correlating to the three carbonyl moieties, and an amide-NH stretch (at 3362 cm^−1^). Finally mass spectrometric analysis showed a parent ion at *m*/*z* = 395.0959 in good agreement with the calculated value. The carboxylic acid 6 was synthesised *via* a similar procedure to that used to generate the acid-functionalised, diaryl ureas 3–5*via* ring opening of succinic anhydride by 3-nitroaniline.^27^ The dicarboxylic acid 7 was synthesised using a previously reported procedure.^14^

### Gelation studies

To probe the self-assembly capabilities of carboxylic acids 1–7, gelation studies were initially undertaken. Hydrogels of the carboxylic acids were generated using the glucono-δ-lactone protocol reported by Adams and co-workers.^28,29^ Initial gelation studies revealed successful hydrogelation of carboxylic acids 1 and 3: indeed we find that 3 is a supergelator, as defined elsewhere,^30^ with a critical gelator concentration of 0.1 wt% (Table 1).

**Table 1:** Gelation properties of 1–7 where; G = gel (withstanding the vial inversion test for > 1 hour)^a^

Molecule	Gelation state	CGC [mM]	wt%
1	G	98.8^b^	2.0
2	GP	—	—
3	G	2.7	0.1
4	P	—	—
5	P	—	—
6	P	—	—
7	G	0.9	0.03

a GP = Gelatinous precipitate, P = precipitate.

b At a concentration of 297.2 mM (6.0 wt%) molecule 1 can also behave as a thermogelator.

The functionalised dicarboxylic acid 7 has already been reported to be a supergelator,^14^ but carboxylic acid 2 was not able to form stable hydrogels which implies that bifunctionality is required to promote supramolecular network growth. Of the previously unreported functionalised carboxylic acids studied (3–6), only the system with the nitro substituent in the *meta* position with respect to the urea bond (3) formed stable gels (as shown by the vial inversion test and rheological analysis; see ESI, Fig. S10†). Rheological analysis undertaken on hydrogels of 3 revealed an increased maximum storage modulus (400 kPa) with respect to gelator 7 (294 kPa: the concentration of both gelators was 20 mM, see ESI, Fig. S11†).^14^ The failure of carboxylic acids 4 and 5 to yield stable gels highlights the importance of the nitro moiety, and its substitution-position on the aromatic ring, in the formation of complementary hydrogen bonding networks. It is proposed that *meta*-substitution of the nitro moiety allows favourable self-assembly towards gelation (as is demonstrated in analogues of 3–6 reported in previous studies),^14–16^ whereas *para* substitution leads to crystallisation and ultimately to precipitation from solution. Furthermore, when the urea moiety was absent, as in the case of the carboxylic acid 6, no fibril growth and hence no gelation was observed. The differences in the assembly of the hydrogelators 3 and 7 were evident from studies on the dye absorption capabilities of each gelator. Whilst gels of compound 7 absorbed aromatic dyes such as methylene blue from aqueous solution (as previously reported), the carboxylic acid 3 did not demonstrate such ability (see ESI, Fig. S12†).^14–16^

### Initial blending procedures

To investigate the potential of network formation within an existing polymer network, films were cast successfully from blends of the carboxylic acids 1–7 and poly(ethylene-*co*-acrylic acid) (15 wt% acrylic acid: pEAA15). Blends were obtained *via* dissolution of both polymer and low molecular weight carboxylic acid in dimethylformamide (DMF) and removal of solvent under high vacuum. In the case of blends of pEAA15 with the diacid 1 it was found that phase separation occurred at an additive loading of 10 wt% as determined by DSC analysis (see ESI, Fig. S13†). In the light of this observation, only those blends containing 1 wt% and 5 wt% of 1 in pEAA15 were investigated in more detail.

### Tensile properties of pEAA15 blends

Mechanical analysis was carried out on blends of pEAA15 and carboxylic acids 1–7 to assess the impact of low molecular weight additives at loading levels of 1 and 5 wt%. Studies were undertaken on films (average dimensions 40 × 10 × 1 mm) using a tensometer, with a true strain rate of 0.2 s^−1^. Each sample was analysed five times and the average stress–strain profile was recorded (see Table 2 and ESI Fig. S14–S20†).

**Fig. 3:**
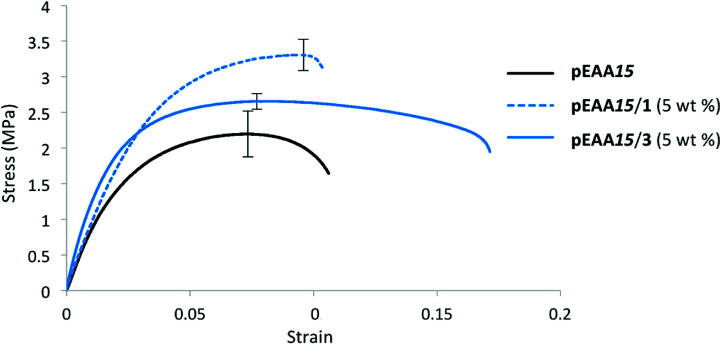
Stress–strain curves (average of five analyses plotted) for pEAA15 (black), pEAA15/1 (5 wt%) (blue dashed), pEAA15/3 (5 wt%) (blue solid).

It was found that blending the carboxylic acids 1 and 2 with pEAA15 had a significant impact upon the polymer's mechanical properties (Table 2). The diacid 1 was found to increase the tensile strength of the bulk phase at low weight concentrations (≤5 wt%) (see Table 2 and Fig. 3). Blending of 1 wt% of the diacid 1 was thus found to afford a stronger but more brittle material than pure pEAA15 as reflected in the increased tensile strength and Young's modulus, though with decreased uniform strain and energy absorbed. At 1 wt% incorporation, compound 1 is thus simply an anti-plasticizer for pEAA15. However, blends comprising 5 wt% of 1 with showed significant increases in both the strain-to-fracture and fracture stress compared with pEAA15 itself, as well as increased tensile strength and Young's modulus. This combination of increased stiffness, toughness, and elasticity of the material at 5 wt% incorporation of dicarboxylic acid 1 indicates that a change in the degree of ordering of the system has occurred (see Fig. 3).^17,31^

**Table 2:** Mechanical properties of films formed of blends of pEAA15 and carboxylic acids 1–7 in 1 and 5 wt% with respect to plasticizer (film dimensions averaging 40 × 10 × 1 mm, true strain rate of 0.2 s^−1^)

Film system	wt% additive	Tensile strength (MPa)	Fracture stress (MPa)	Uniform strain (%)	Strain to fracture (%)	Energy absorbed (MPa)	Young's modulus (MPa)
pEAA15		2.20	1.95	7.30	9.84	0.26	117.47
pEAA15/1	1	2.83	2.75	3.26	3.59	0.11	220.49
	5	3.30	3.12	9.54	10.38	0.39	123.78
pEAA15/2	1	1.86	1.49	9.09	9.58	0.13	23.72
	5	0.95	0.46	62.80	69.50	0.34	0.98
pEAA15/3	1	2.28	2.09	8.00	10.66	0.26	117.48
	5	2.66	1.95	9.21	17.14	0.41	146.21
pEAA15/4	1	1.81	1.37	12.90	17.62	0.27	54.45
	5	1.85	1.67	5.52	6.13	0.08	58.42
pEAA15/5	1	2.04	1.72	16.26	18.37	0.31	52.03
	5	0.36	0.26	5.72	6.49	0.02	24.22
pEAA15/6	1	1.75	1.54	13.14	16.44	0.23	18.53
	5	0.59	0.32	16.10	20.40	0.09	6.57
pEAA15/7	1	1.90	1.78	12.91	14.88	0.22	53.04

### Strain

In contrast to the increase in stiffness resulting from incorporation of dicarboxylic acid 1 with pEAA15, the monocarboxylic acid 2 acted as a plasticizer when blended at sufficient loading levels. Thus, at a loading of 5 wt% of 2, a 70-fold increase in strain-to-fracture was thus observed as well as a decrease in both the tensile strength and Young's modulus. It is proposed that such plasticization results from the inability of the monocarboxylic acid 2 to effectively self-assemble (beyond hydrogen bonded dimerisation), preventing the formation of effective reinforcing networks within the polymer blends,^17,30^ (see gelation studies, Table 1).

Blending of the urea-functionalised carboxylic acid 3 with pEAA15 afforded an increase in the material's tensile strength as well as its uniform strain (Table 2 and Fig. 3). The increase in tensile strength was less pronounced than in the blends with the dicarboxylic acid 1, but in contrast the increase in uniform strain was greater. It is proposed that these changes in mechanical properties are a result of the introduction of a weaker secondary interaction in the self-assembly motif of the bisarylnitro urea moiety in 3 (Fig. 1C).

The importance of the presence and substitution-position of the nitro functionality (as well as a second aromatic ring and urea moiety) in reinforcing the properties of the bulk polymeric phase was confirmed by analysis of blends of carboxylic acids 4–6 with pEAA15. In these studies it was found that these additives acted solely as plasticizers, lowering the tensile strength of the polymer and increasing the uniform strain (Table 2). Thus, blending the carboxylic acids 4–6 did not have the same beneficial effects on mechanical properties as found in the blends of pEAA15 with 3. The importance of the interactions between the carboxylic acid moieties of copolymer and additive was also demonstrated *via* analysis of blends of the gelator 7 with pEAA15. At a loading of just 1 wt%, additive 7 was found to enhance the properties of the copolymer substantially with respect to both tensile strength and Young's modulus (Table 2).

### Differential scanning calorimetric studies

In order to characterise the thermal transitions of the blends DSC analysis was conducted. Studies were focused on the blends that demonstrated modified mechanical properties (Table 2) when compared to the bulk polymer, in accordance with the model shown in Fig. 1. The transition temperatures were determined by the application of two successive heat/cool cycles, the first cycle to remove thermal/processing histories and the second to identify the transitions (*e.g. T*_g_ and *T*_m_) (see ESI Fig. S21–S24†).

Each blend demonstrated plasticization as evidenced by the observation of lower *T*_g_, and *T*_m_ values with respect to pEAA15 (Table 3). Interestingly a slight increase in *T*_g_ was observed in the blends containing higher additive loadings (5 wt%) in comparison to the blends featuring 1 wt% of the carboxylic acid additives 1 and 3. This trend was, however, not observed in the case of additive 2. It was noted that transition temperatures observed for random copolymer pEAA15 were in good agreement with previously-reported value.^32^ Percentage crystallinity (calculated from comparison with the enthalpy of melting of crystalline poly[ethylene])^32^ showed little difference between the pure copolymer and blends of the copolymer and the bifunctionalised carboxylic acids 1 and 3 (Table 3). Only those samples blended with the monofunctionalised acid 2 showed any slight decrease in percentage crystallinity. It is therefore concluded that the small molecule additives do little to decrease the overall crystallinity of the copolymer, suggesting that the interactions are indeed carboxylic acid polymer-carboxylic acid additive specific.^25^

**Table 3:** Thermal properties of formed of blends of pEAA15 and additives 1–3 in 1 and 5 wt% with respect to plasticizer after the second heat/cool cycle (heating rate 15 °C min^−1^, cooling rate 5 °C min^−1^) with percent crystallinity (*χ*) determined by assuming a melt enthalpy of 293 J g^−1^ for 100% crystalline poly(ethylene)^32^

Film system	wt% additive	*T*_g_	*T*_m_	*T*_c_	*χ*
pEAA15		−12	84	73	7
pEAA15/1	1	−14	82	71	7
	5	−13	82	71	7
pEAA15/2	1	−15	82	72	5
	5	−15	81	71	4
pEAA15/3	1	−16	82	72	7
	5	−15	83	72	6

Additional DSC studies were undertaken whereby two individual heal/cool cycles (on the same sample) were separated by a 24 hour gap (20 °C) (ESI, Fig. S25–S28†). This gap was introduced to probe the ability of the blends to relax into more thermally stable states over a period of 24 hours. Each of the blends was found to exhibit identical thermal characteristics to those observed 24 hours previously (ESI, Fig. S25–S28†). Several lower thermal transitions (*ca.* 7–10 °C), not observed in the second heat/cool cycles (see Table 3) were apparent in both the first scans of the individual heal cool cycles separated by 24 hours. Interestingly such lower thermal transitions evident in the DSC thermograms of polymer blends of pEAA15/1-3 (recorded from the post relaxation analysis) correlate well with those observed in well-defined poly(ethylene-*co*-acrylic acid) block copolymers.^25^

### Healing studies

Healing studies were undertaken on pEAA15 and its blends using mechanical property recovery as the key indicator. It was decided to limit these studies to the blends that possessed improved properties (with respect to tensile strength and uniform strain) when compared to the bulk polymer. For this reason, blends of carboxylic acids 1 and 3 with pEAA15 at an additive loading of 5 wt% and 2 with pEAA15 (1 wt%, selected as a control with limited weakening effects) were studied (Table 4 and ESI Tables S1–S3†).

Three independent studies were undertaken on films of the blends (40 × 10 × 1 mm) to ascertain the degree of healability. For the thermal studies, films of blends were cast from solution (DMF) and cut into two pieces using a scalpel before being placed in contact without overlap of the cut edges. The films were then held at 60 °C for 2 hours (entries a in Table 4) and 50 °C for 8 hours (entries b in Table 4). These temperatures were chosen as they are at least 15 °C lower than any of the endotherms recorded in the DSC analysis.

**Table 4:** Percentage healing of films of the blends based on tensile strength, energy absorbed and Young's modulus recovery^a^

Film system	wt% blended	Tensile strength recovery (%)			Energy absorbed recovery (%)			Young's modulus recovery (%)		
		a	b	c	a	b	c	a	b	c
pEAA15	—	5.5	17.7	45.5	0.8	3.1	23.1	4.8	12.1	22.4
pEAA15/1	5	2.7	—	41.0	0.3	—	25.6	10.6	—	17.6
pEAA15/2	1	30.6	38.7	10.8	10.0	23.1	5.4	97.9	75.8	78.4
pEAA15/3	5	33.1	42.1	73.3	3.2	5.1	34.1	12.7	12.3	28.3

a (a) Healing at 50 °C 8 hours, (b) healing at 60 °C 2 hours and (c) healing under pressure (0.98 MPa) 8 hours. The stress–strain data are reported in the ESI, see Fig. S29–S39.

It was found that samples of pure pEAA15 demonstrated only limited healability (Table 4). Blends of pEAA15 and carboxylic acid 3 were shown to dramatically increase the thermal healability of pEAA15 (for example a 28% increase in recovery of tensile strength with respect to the pure copolymer, Table 4). Although the blend featuring carboxylic acid 2 exhibited a greater degree of healing with respect to energy absorbed and Young's modulus recovery there was a drop in the mechanical properties when compared to pure pEAA15. This blend also showed decreases in the thermal transition temperatures which in turn implies simple plasticisation of the bulk polymer phase.

Notably, the thermal healing studies revealed the ability of the carboxylic acid 3 blended with pEAA15 to heal with greater efficiency (with respect to each mechanical property recorded, Table 4) than the blend formed with the dicarboxylic acid 1. These data suggest that the insertion of a secondary supramolecular functionality (in the form of the diaryl urea of 3, see Fig. 1), which is capable of self-assembly to form an alternative network, actually increases the healability of the bulk phase without disrupting its mechanical performance.

An additional healing study on these blends involved the application of pressure to the cut films. Films were prepared (40 × 10 × 1 mm) and cut with a scalpel before the cut edges were overlapped (1 mm lengthways) and then subjected to a pressure of 0.98 MPa overnight. The films containing the carboxylic acid 3 additive healed most successfully under this regime using tensile strength recovery as the key indicating factor (see entries c in Table 4). Interestingly the plasticized blends of pEAA15/2 exhibited some limited recovery when this healing method was used, in terms of recovering both the tensile strength and energy absorbed to break.

Further healing studies were conducted with poly(ethylene-*co*-acrylic acid) having 5% (pEAA5) and 20% (pEAA20) acrylic acid content, blended with the additives 1 and 3. DSC analysis of the blends of pEAA5 with carboxylic acid 1 revealed phase separation (observed *via* melt point of the additive) at additive loadings of 5 wt% in contrast to those of pEAA20 which are homogeneous at 10 wt% (see ESI Fig. S40–S41†). This trend is in agreement with the proposal that the small molecule additives interact with the carboxylic acid moieties present in the polymeric backbone.

**Fig. 4:**
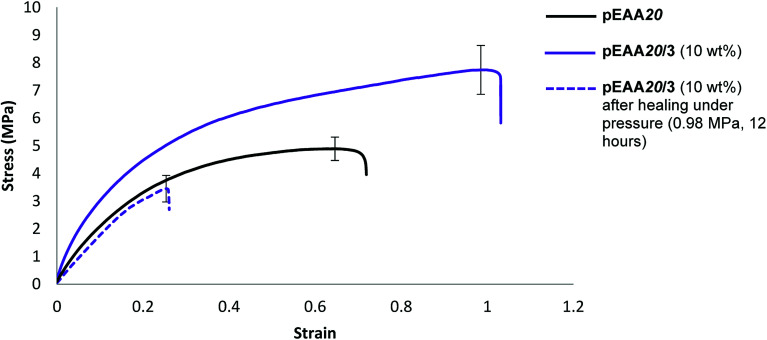
Stress strain curves (average of five analyses plotted) showing pEAA20 and pEAA20/3 before (solid purple) and after (dashed purple) after healing under pressure (0.98 MPa) (8 hours). *Note that pEAA20 failed to heal under similar pressure conditions.

To probe potential system hardening and healability, films of the blends formed between pEAA20 and carboxylic acids 1 and 3 (each at an additive loading of 10 wt%) were subjected to tensile testing (ESI Fig. S42–S45†). An increase in strength and elastomeric response was realised in both of these blends (see Table 5 and Fig. 4).

**Table 5:** Mechanical properties of films formed from blends of pEAA20 and carboxylic acids 1 and 3 at additive loadings of 10 wt% (film dimensions averaging 40 × 10 × 1 mm, true strain rate of 0.2 s^−1^)

Film system	wt% additive	Tensile strength (MPa)	Fracture stress (MPa)	Uniform strain (%)	Strain to fracture (%)	Energy absorbed (MPa)	Young's modulus (MPa)
pEAA20		4.86	4.47	64.7	72.1	2.84	26.79
pEAA20/1	10	4.23	4.11	122.9	129.8	4.85	40.24
pEAA20/3	10	6.30	5.78	98.4	103.1	4.90	33.76

Studies on the healability of blends of pEAA20 focused on a comparison of pEAA20 with the best performing blend (see Table 5). The blend pEAA20/3 (10 wt%) was the most successful under pressurised healing conditions (Table 4) using the healing conditions described above. Under these conditions recovery of the properties was not observed in pEAA20 whilst the blends of pEAA20 and 3 (10 wt%) exhibited a degree of healability in terms of tensile strength (55% recovery), energy absorbed to break (17% recovery) and Young's modulus (54% recovery) (see Fig. 4).

## Conclusions

It has been demonstrated that blending functionalised molecular additives with copolymers of ethylene and acrylic acid can serve the dual purpose of both reinforcement and increased healability *via* the creation of a supramolecular ‘network within a network’. This was realised by generating a soft ‘gelator type’ network phase within a polymeric network. This scenario is only possible when the small molecular additives are able to interact by hydrogen bonding with the residues within the copolymer structure that are responsible for supramolecular bonding and network formation. Manipulation of the mechanical properties of the bulk polymer phase has been demonstrated by varying the additive concentrations as well as through changes in the structural composition of the additive. It was noted that those additives with the nitro moiety in the *meta* position provide the most beneficial additive properties to the resulting blends.

It is proposed that the introduction of functionalised small molecule additives to reinforce and promote healability could be developed for a range of copolymers. Variations in the moieties responsible for the formation of soft ‘gelator type’ networks would enable control over structural stability whilst variations in the moieties responsible for additive–polymeric interactions would enable compatibility. However, the composition of the copolymer is also crucial: it must feature complementary residues to permit binding with the low molecular weight additive. Furthermore the crystalline and amorphous structures of the copolymer must allow self-assembly of the low molecular weight additive if beneficial properties (such as recovery of strength on healing) are to be realised.

## Experimental

Chemicals and solvents were purchased from Sigma Aldrich and used as purchased, unless otherwise specified. THF was distilled from sodium and benzophenone under inert conditions prior to use. NMR spectra were obtained using Bruker Nanobay 400 and Bruker DPX 400 instruments (operating at 400 MHz and 100 MHz for ^1^H NMR and ^13^C NMR spectroscopy, respectively). NMR samples were prepared in DMSO-*d*_6_ or CD_3_OD and dissolution was achieved with slight heating and sonication (5–10 minutes). Mass spectra were obtained on a Thermo-Fisher Scientific Orbitrap XL LCMS (operating in electrospray mode) – samples were prepared in DMSO for direct injection (1 mg mL^−1^). A Perkin Elmer 100 FT-IR (diamond ATR sampling attachment) was employed for IR spectroscopic analysis. Samples for IR characterisation were in powder form. UV spectra were recorded using a Varian Cary 300 Bio or a PerkinElmer Lambda 25 UV/Vis spectrometer. Sample solutions were analysed in quartz cuvettes with a 5.0 mm path length and were baseline corrected with respect to a blank cell with the appropriate solvent.

The pH-stimulated hydrogels of 1 and 3 were produced *via* dissolution of the gelator in NaOH_(aq.)_ (0.5 mL, 0.1 M) followed by addition of glucono-δ-lactone (0.5 mL, 0.2 M).^26^ The solutions were then left for 2 hours to acidify and gel. The critical gelation concentration (CGC) of 3 was determined in a 2 mL screw top glass vial. Minimum gelator mass was determined to the nearest 1 mg, then varied every 0.2 mg to obtain increased accuracy of the CGC value. Dye uptake measurements were carried out *via* extraction of 0.5 mL sample from dye/gelator mixtures, filtering through sterile syringe filters (0.45 μm Minisart® syringe filter).

Thermogravimetric analysis employed a TA Instruments TGA Q50 attached to a TGA heat exchanger, platinum crucible and an aluminium TA-Tzero pan (heating rate 15 °C min^−1^ to 500 °C). Differential scanning calorimetry analysis employed a TA DSC Q2000 with TA Refrigerated Cooling System 90 (aluminum TA-Tzero pans and lids) (heating rate 15 °C min^−1^, cooling rate 5 °C min^−1^).

The strengths of films were determined by texture analysis (Texture Analyser, Stable Microsystems). Analysis was conducted with A/TG tensile grips (screw-initiated vice clamp, knurled-jaw faces 35 mm × 35 mm) at a true strain rate of 0.2 s^−1^, using a trigger force of 0.01 N. The strength of the film was taken as the maximum force applied before fracture which is seen graphically as a sharp drop in recorded force. All films tested were prepared by solvent casting (DMF) to facilitate film homogeneity. It was later ascertained that tensile properties were equivalent for melt and solvent cast films of pEAA15/1. Films were cut into a dog-bone shape before testing, giving a strain-measurement region with dimensions averaging 40 × 10 × 1 mm.

Carboxylic acids 1 and 2 were used as supplied. The small molecule additives 3–5 were synthesised according to the following procedure. The appropriate aromatic bis amine, 1-(4-aminophenyl)-3-(3-nitrophenyl)urea (to give 3) or 1-(4-aminophenyl)-3-(4-nitrophenyl)urea (to give 4) (0.1 g, 0.36 mmol) or 1-(4-aminophenyl)-3-phenylurea (to give 5) (0.08 g, 0.36 mmol) was dissolved in dry THF (50 mL). To this solution succinic anhydride (0.032 g, 0.36 mmol) was added directly and the solution was then stirred under reflux for 24 hours. The product was precipitated into HCl_(aq)_ (1.0 M, 200 mL), collected *via* filtration at the pump and then washed with H_2_O (2 × 25 mL) before drying *in vacuo* (80 °C, 2 hours) to afford:-

(3) 4-((4-(3-(3-Nitrophenyl)ureido)phenyl)amino)-4-oxobutanoic acid, a light brown powder, (0.12 g, 86%) *T*_dec_ 252 °C; IR (ATR)/cm^−1^ 3362, 3275, 1696, 1671, 1655, 1599, 1554, 1515, 1403, 1348, 1255, 1202, 1173, 1047, 888, 839, 795, 722; ^1^H NMR (400 MHz, DMSO-*d*_6_) = 9.89 (s, 1H), 9.39 (s, 1H), 8.97 (s, 1H), 8.58 (m, 1H), 7.80 (m, 1H), 7.73 (m, 1H), 7.51 (m, 3H) 7.39 (m, 2H) ppm; ^13^C NMR (100 MHz, DMSO-*d*_6_) = 175.8, 169.7, 153.8, 149.1, 140.2, 135.3, 135.1, 130.9, 124.6, 121.9, 119.9, 114.3, 111.2, 31.3, 29.1 ppm; MS (ESI) *m*/*z* [M + Na^+^] calculated for C_17_H_16_O_6_N_4_Na = 395.0962, found 395.0959.

(4) 4-((4-(3-(4-Nitrophenyl)ureido)phenyl)amino)-4-oxobutanoic acid; a yellow powder, (0.08 g, 61%) *T*_dec_ 255 °C; IR (ATR)/cm^−1^ 3369, 3282, 3056, 1657, 1604, 1553, 1498, 1404, 1324, 1220, 1110, 834, 746, 645; ^1^H NMR (400 MHz, DMSO-*d*_6_) = 9.92 (s, 1H), 9.39 (s, 1H), 8.82 (s, 1H), 8.18 (m, 2H), 7.69 (m, 2H), 7.50 (m, 2H), 7.37 (m, 2H) ppm; ^13^C NMR (100 MHz, DMSO-*d*_6_) = 173.9, 169.8, 151.9, 146.4, 140.9, 134.3, 134.0, 125.1, 119.5, 119.1, 117.4, 30.9, 28.8 ppm; MS (ESI) *m*/*z* [M + H^+^] calculated for C_17_H_17_O_6_N_4_ = 373.1143, found 373.1142.

(5) 4-Oxo-4-((4-(3-phenylureido)phenyl)amino)butanoic acid; a white powder, (0.09 g, 73%) *T*_dec_ 242 °C; IR (ATR)/cm^−1^ 3314, 3270, 3030, 1696, 1638, 1601, 1562, 1444, 1404, 1301, 1226, 1183, 1054, 902, 799, 735, 619; ^1^H NMR (400 MHz, DMSO-*d*_6_) = 12.13 (s, 1H), 9.87 (s, 1H), 8.62 (s, 1H), 8.57 (s, 1H), 7.50 (m, 4H), 7.36 (m, 2H), 7.26 (m, 2H), 6.95 (m, 1H) ppm; ^13^C NMR (100 MHz, DMSO-*d*_6_) = 173.9, 169.6, 152.5, 139.8, 128.7, 121.7, 119.5, 119.0, 118.6, 118.1, 30.9, 28.9 ppm; MS (ESI) *m*/*z* [M + H^+^] calculated for C_17_H_18_O_4_N_3_ = 328.1292, found 328.1290.

(6) 4-((3-Nitrophenyl)amino)-4-oxobutanoic acid; 3-nitroaniline (0.1 g, 0.72 mmol) was dissolved in dry THF (50 mL). To the solution succinic anhydride was added (0.064 g, 0.72 mmol) and the solution stirred under reflux for 24 hours. The product was precipitated into HCl_(aq)_ (1.0 M, 200 mL), the precipitate collected by filtration and washed with H_2_O (2 × 25 mL) to yield the title compound as a yellow powder (0.142 g, 83%) *T*_dec_ 239 °C; IR (ATR)/cm^−1^ 3260, 3198, 3105, 2863, 2567, 1694, 1674, 1610, 1553, 1523, 1432, 1403, 1340, 1256, 1176, 1083, 951, 806, 732, 670; ^1^H NMR (400 MHz, DMSO-*d*_6_) = 10.47 (s, 1H), 8.63 (s, 1H), 7.87 (m, 2H), 7.58 (m, 1H), 2.60 (t, 2H, *J* = 6.8 Hz), 2.52 (t, 2H, *J* = 6.8 Hz) ppm; ^13^C NMR (100 MHz, DMSO-*d*_6_) = 173.7, 170.9, 147.9, 140.3, 130.1, 124.8, 117.5, 112.9, 31.0, 28.5 ppm; MS (ESI) *m*/*z* [M + H^+^] calculated for C_10_H_11_O_5_N_2_ = 239.0668, found 239.0670.

## Conflicts of interest

There are no conflicts to declare.

## Supplementary Material

RA-008-C8RA09597C-s001
