# Supplementary material for: Inducing hardening and healability in poly(ethylene-co-acrylic acid) via blending with complementary low molecular weight additives
Source: RSC Adv. 2018 Dec 12;8(72):41445–53. doi: 10.1039/c8ra09597c (PMC9091865; doi:10.1039/c8ra09597c)
Supplement: RA-008-C8RA09597C-s001 [file RA-008-C8RA09597C-s001.pdf]

Electronic Supplementary Material (ESI) for RSC Advances.
This journal is © The Royal Society of Chemistry 2018

Inducing hardening and healability in poly(ethylene-*co*-acrylic acid) via blending with complementary low molecular weight additives

Benjamin C. Baker[a]; I. German[b]; Gary C. Stevens[b]; Howard M. Colquhoun[a]; and Wayne Hayes [a]*

[a] Department of Chemistry, University of Reading, Whiteknights, Reading, RG6 6AD, UK. Email: w.c.hayes@reading.ac.uk, Telephone: +44 118 378 6491, Fax: +44 118 378 6331
[b] Gnosys Global Ltd., 17-18 Frederick Sanger Road, The Surrey Research Park, Guildford, Surrey, GU2 7YD, UK

Supplementary Information

**Contents**		**Page**
**Scheme S1**	Synthetic route to compounds **3-5**.	**S1**
**Figures S1-S9**	^{1}H and ^{13}C NMR spectra of **3-6** in DMSO-d_6 or CD_3OD plus IR spectra.	**S2**
**Figures S10-S12**	Gelator **3**: rheology and UV-vis absorption characteristics.	**S9**
**Figure S13**	DSC data for **pEAA*15*/1** (10% wt).	**S11**
**Figures S14-S20**	Stress-strain curves for **pEEA*15*** and additives **1-7** (1/5% wt).	**S11**
**Figures S21-S24**	DSC thermograms for **pEAA*15*/1-3** (0, 1 and 5% wt).	**S15**
**Figures S25-S28**	Relaxation DSC scans of **pEEA*15*/1-3** (0, 1 and 5% wt).	**S16**
**Tables S1-S3**	Tensile properties of **pEAA*15*** and **pEEA*15*/1-3** after healing.	**S18**
**Figures S29-S39**	Stress-strain curves for **pEAA*15*** and **pEEA*15*/1-3** after healing.	**S19**
**Figures S40-S41**	DSC thermograms of **pEAA*5*** and **pEAA*20*** with **1** and **3**.	**S24**
**Figures S42-S45**	Stress strain curves for **pEAA*20*** with **1** and **3.**	**S25**

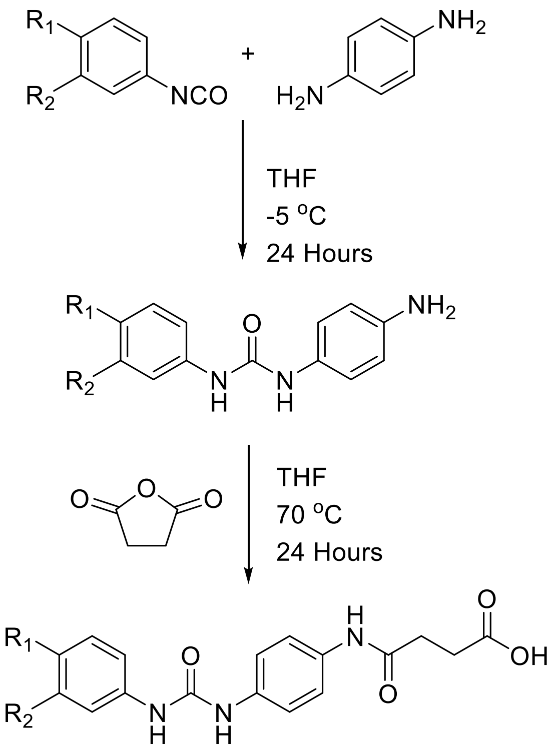

Scheme S1; Generic synthesis of compounds **3-5** (**3**: R_1 = H, R_2 = NO_2. **4**: R_1 = NO_2, R_2 = H. **5**: R_1 = H, R_2 = H).

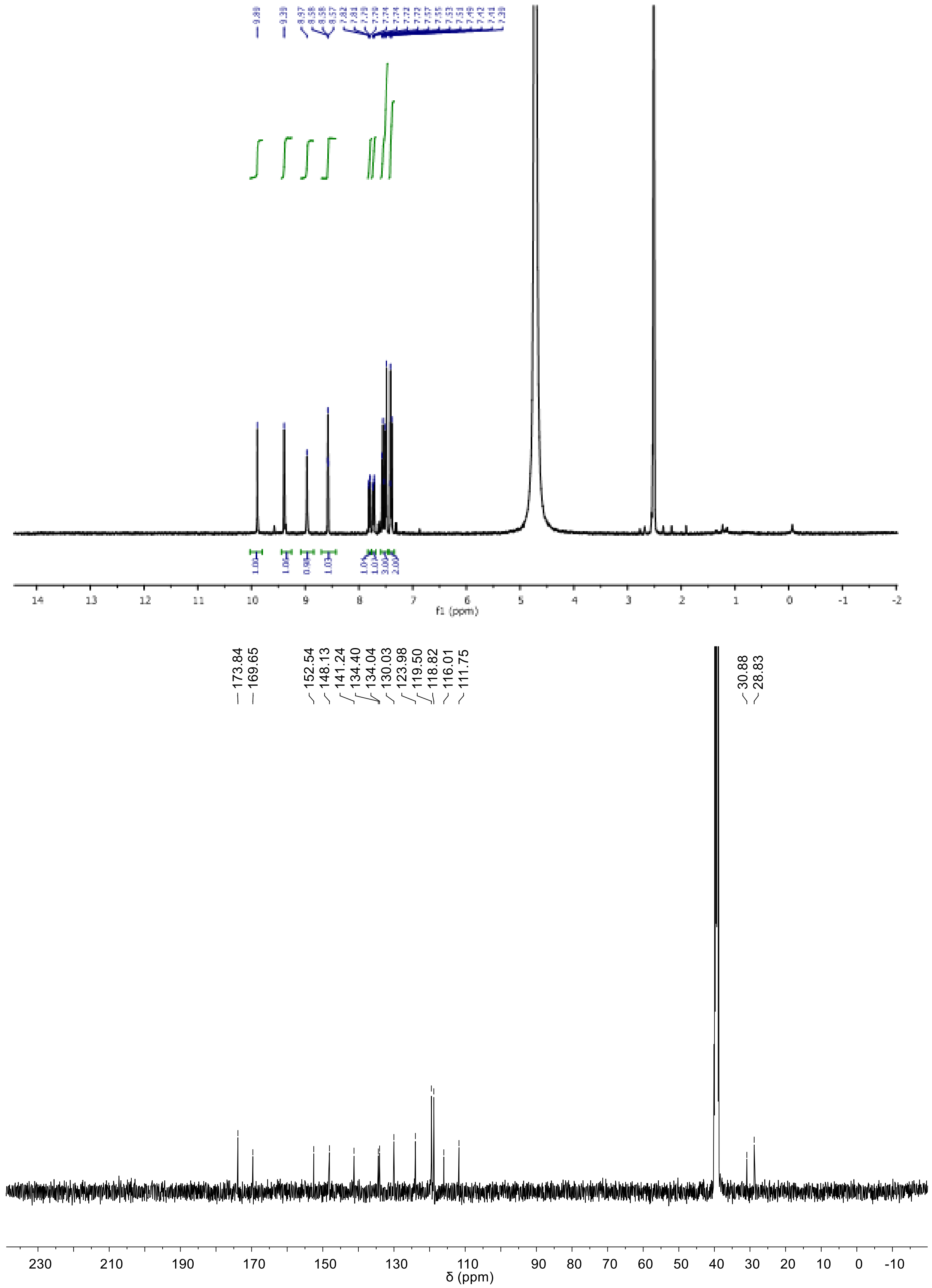

Figure S1; ^{1}H and ^{13}C NMR spectra of **3** in DMSO-d_6

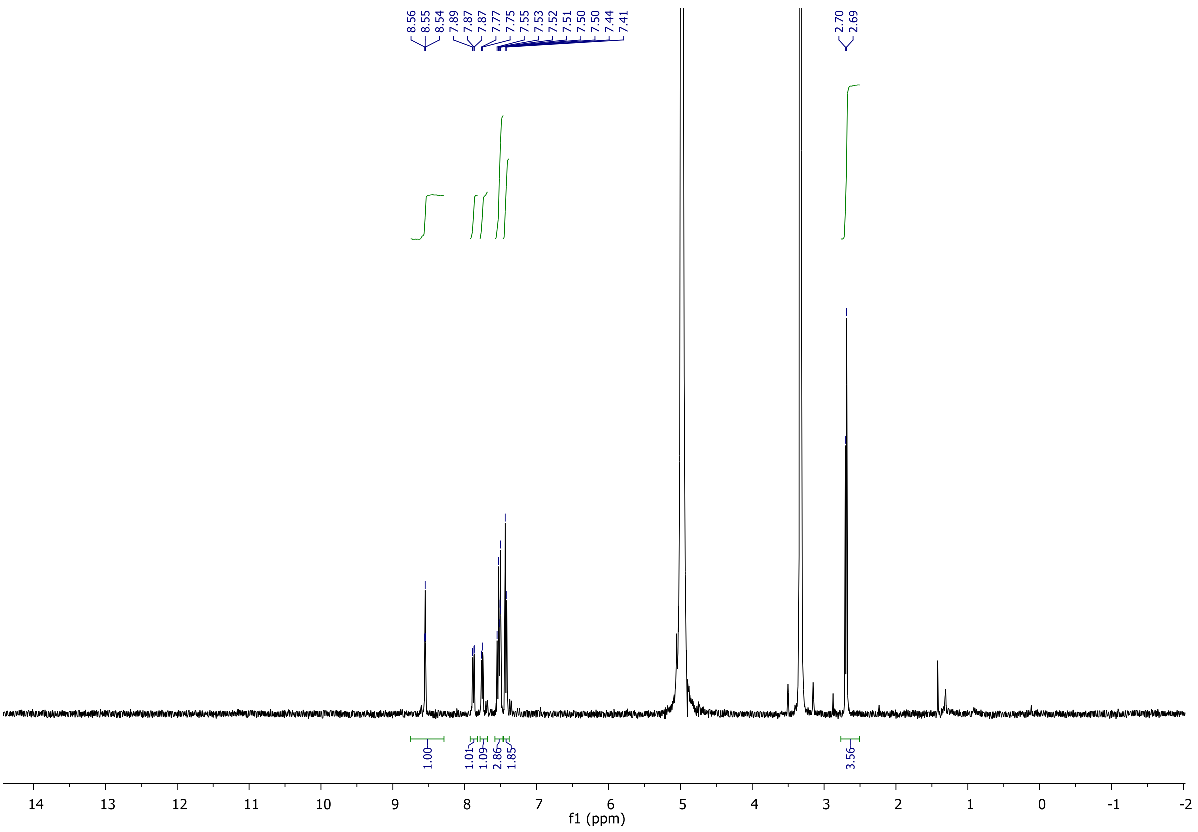

Figure S2; ^{1}H NMR spectrum of **3** in CD_3OD

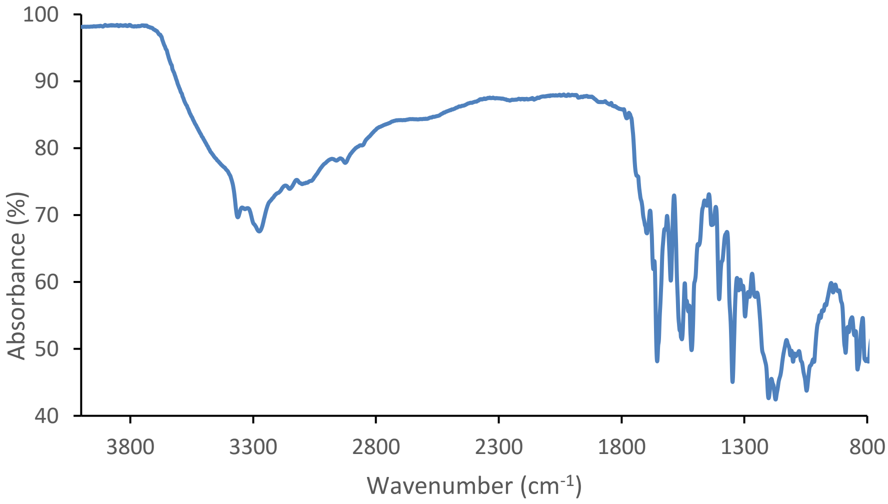

Figure S3; IR spectra of **3**

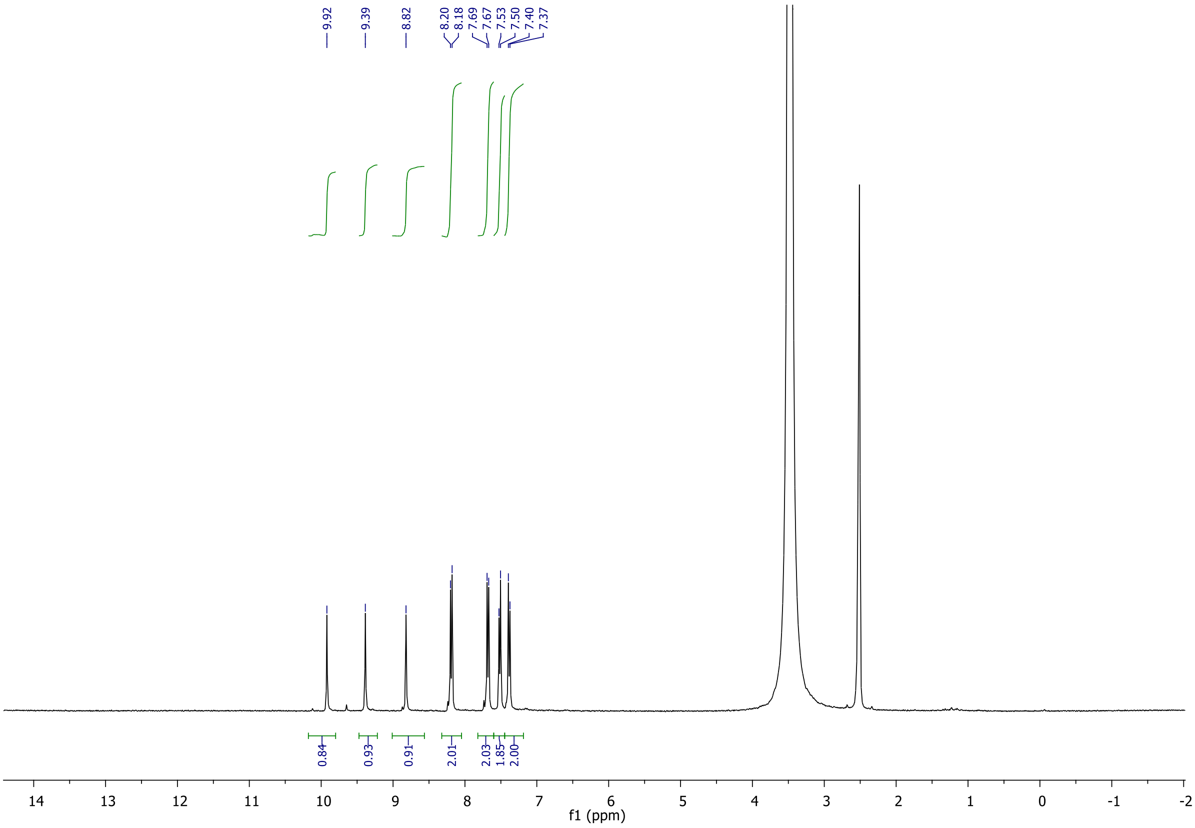

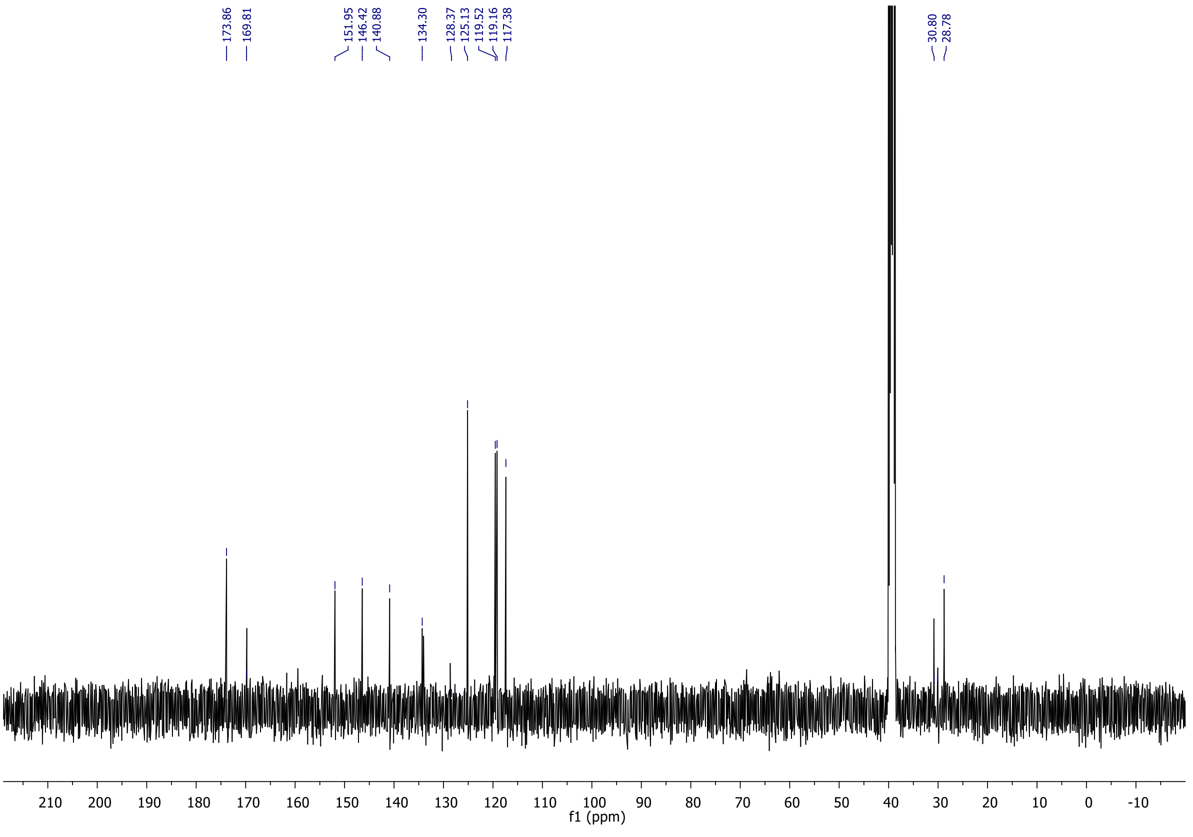

Figure S4; ^{1}H and ^{13}C NMR spectra of **4** in DMSO-d_6

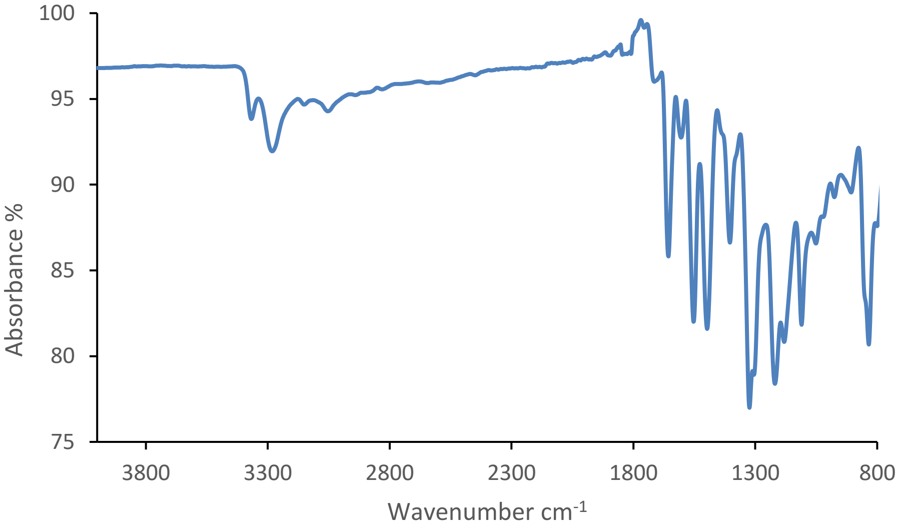

Figure S5; IR spectra of **4**

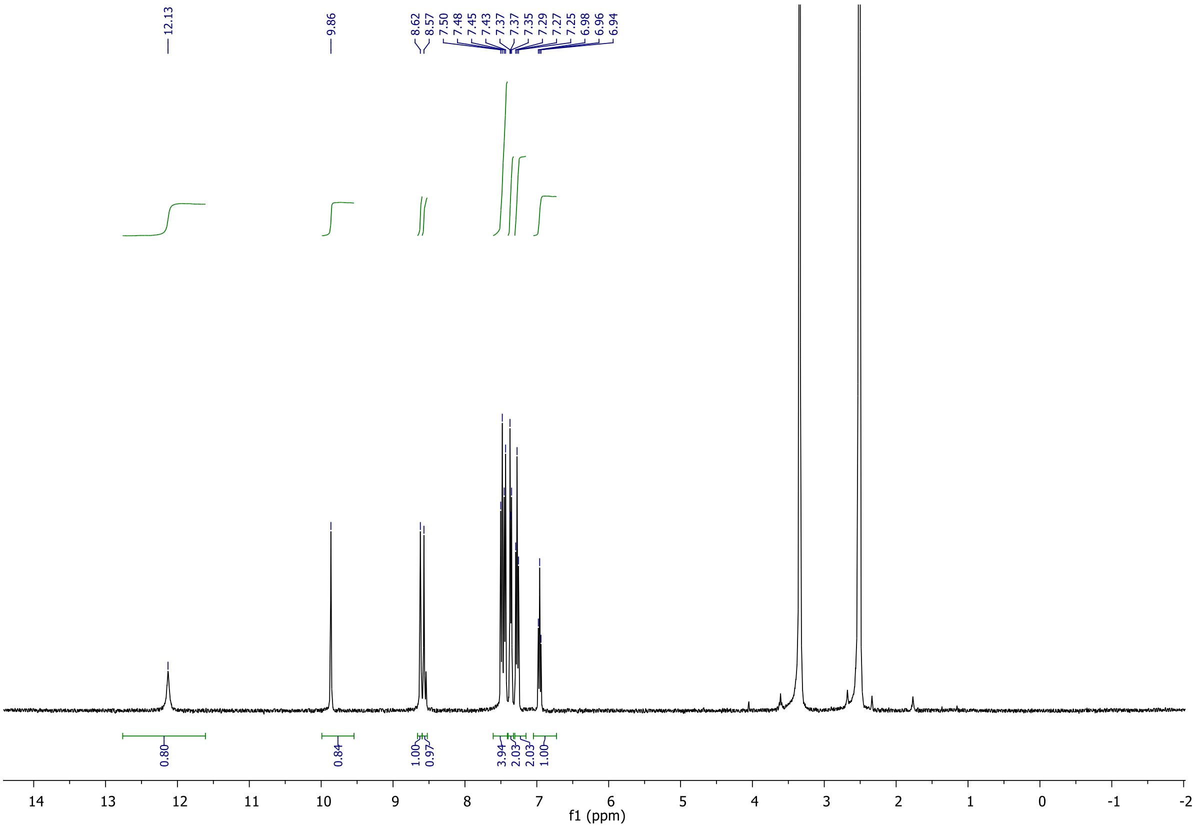

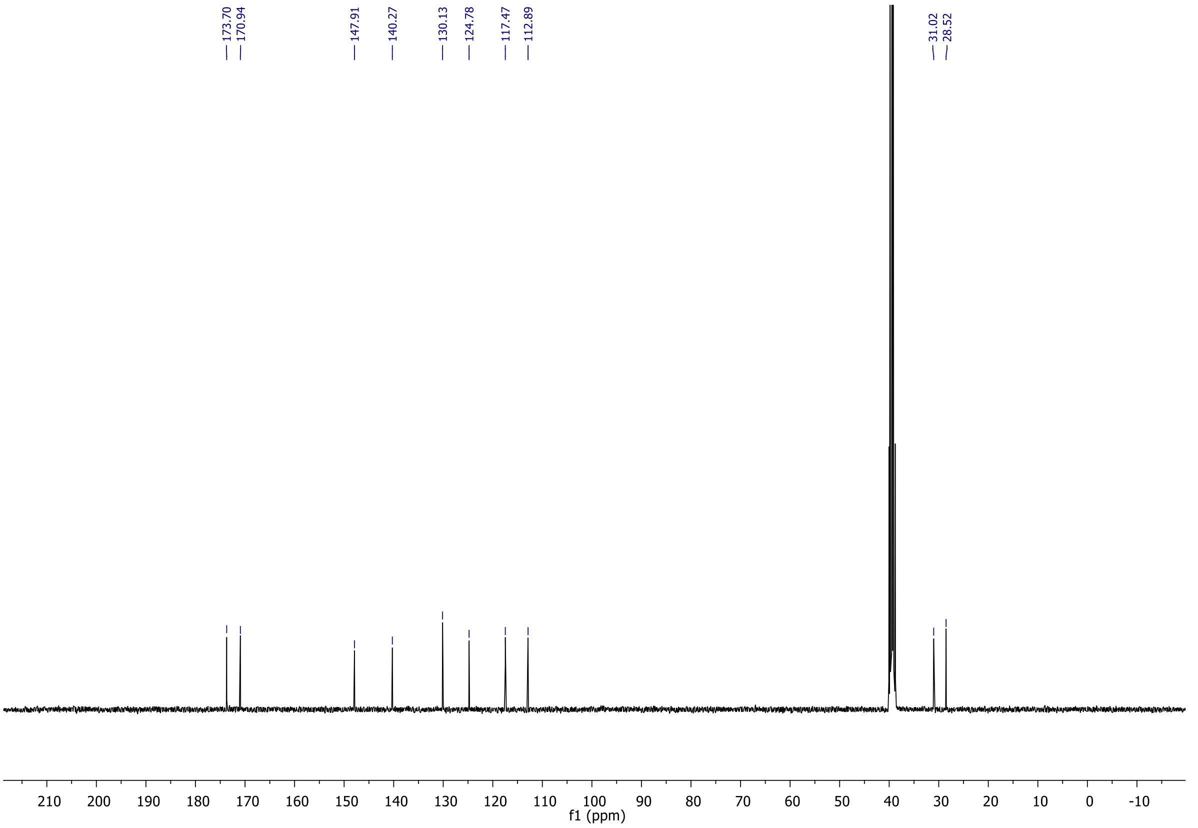

Figure S6; ^{1}H and ^{13}C NMR spectra of **5** in DMSO-d_6

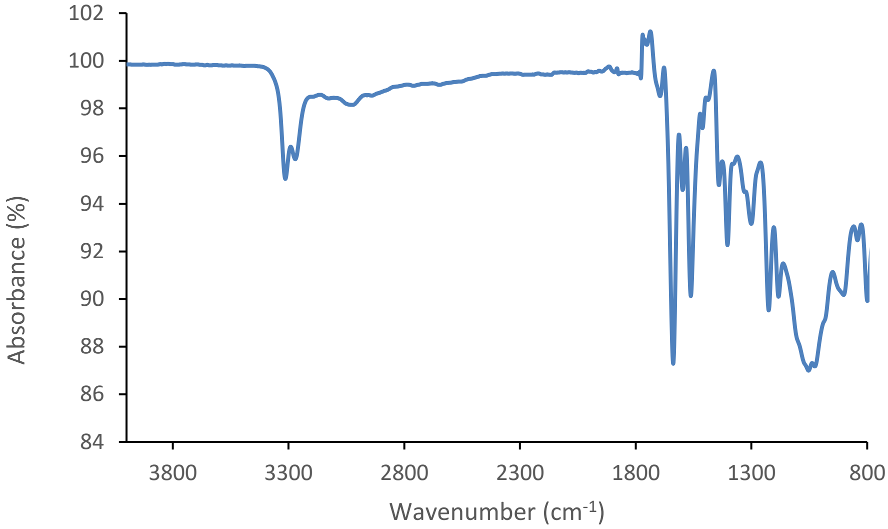

Figure S7; IR spectra of **5**

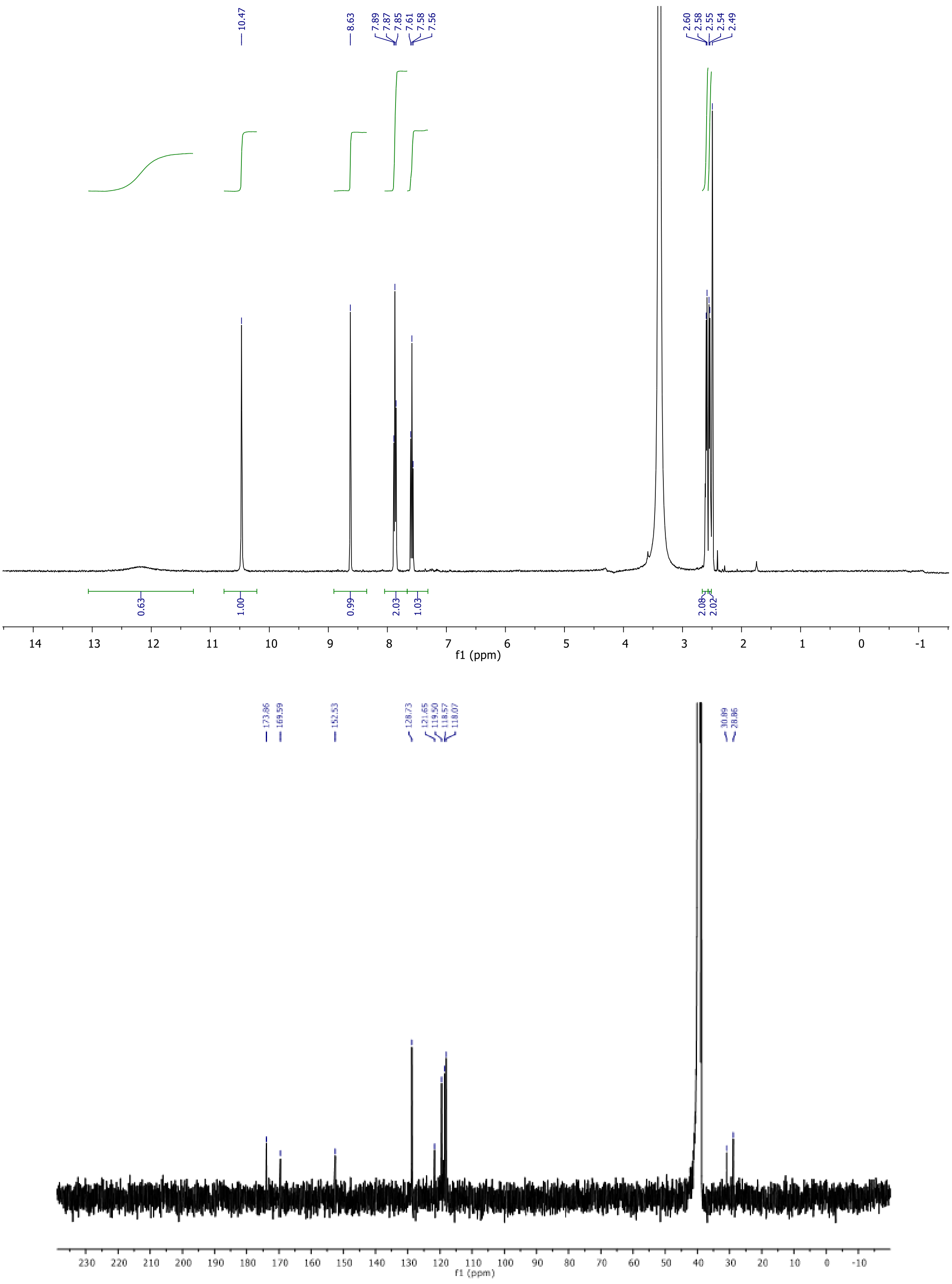

Figure S8; ^{1}H and ^{13}C NMR spectra of **6** in DMSO-d_6

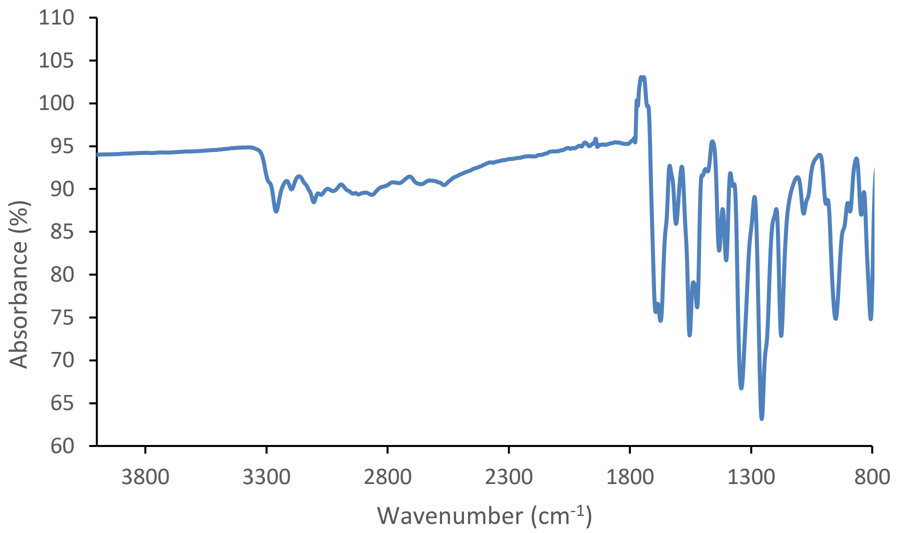

Figure S9; IR spectra of **6**

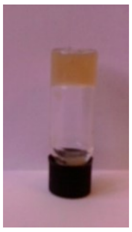

Figure S10; Hydrogelator **3**, CGC 2.7 mM (0.1%wt)

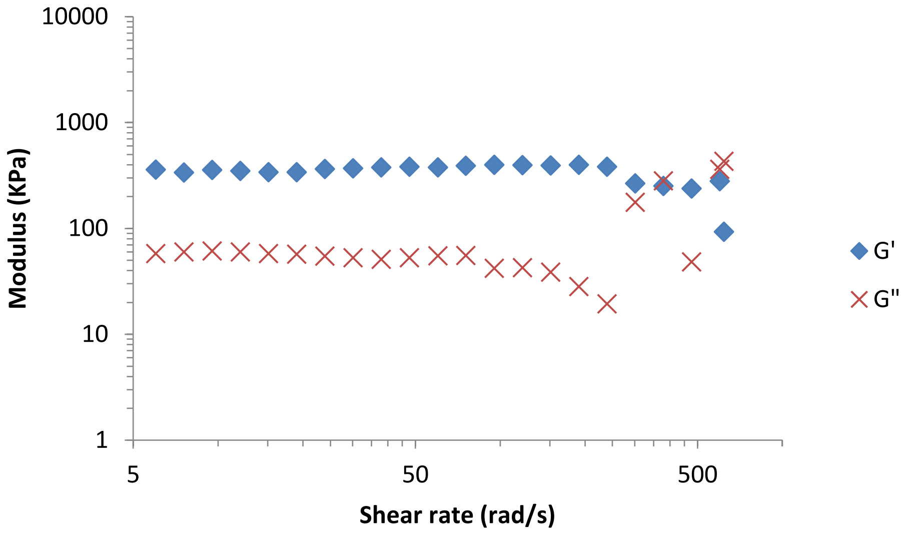

Figure S11; Rheology of aqueous gel of hydrogelator **3** (20 mM).

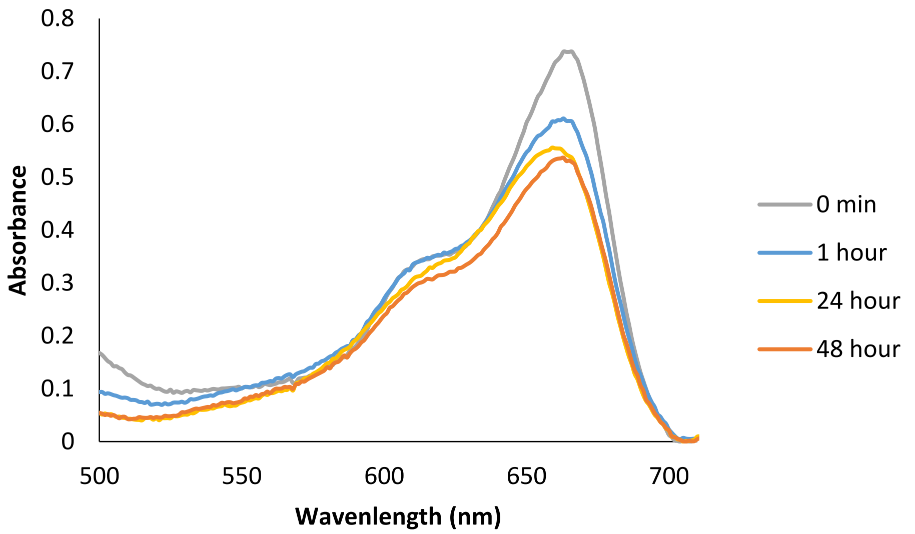

Figure S12; UV/vis absorption spectra of stirred solution of aqueous methylene blue (250 mL, 8 mg L^{-1}) after addition of hydrogelator **3** (1 mL, 80 mM).

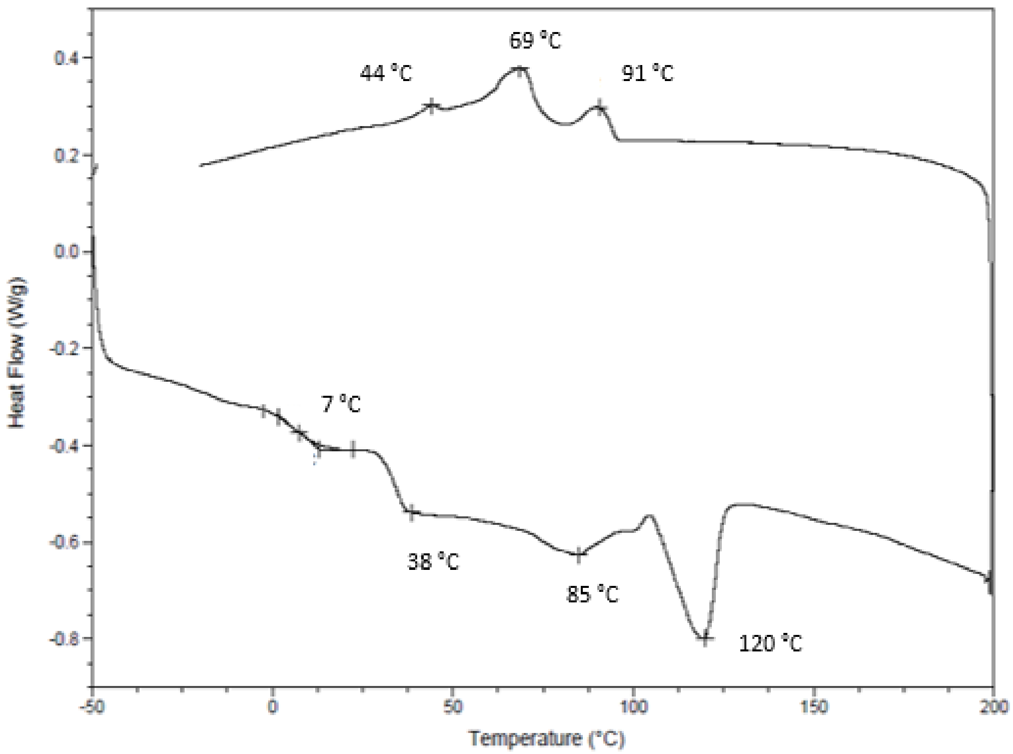

Figure S13; DSC heating (lower) and cooling (upper) curves for **pEAA*15* /1** (10% wt) showing phase separation of polymer and dicarboxylic acid **1** (represented by the melting transition at 120 °C).

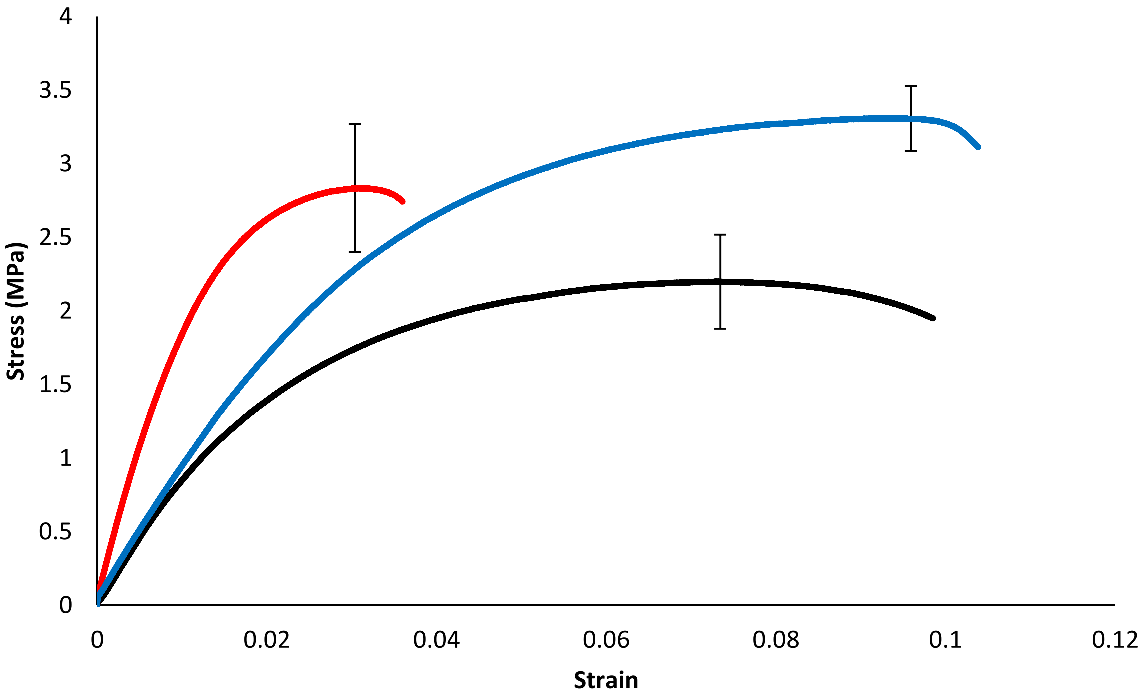

Figure S14; Stress strain curves (average of five samples) for; **pEEA*15*** (black), **pEEA*15*/1** (1% wt.) (red) and **pEEA*15*/1** (5% wt.) (blue).

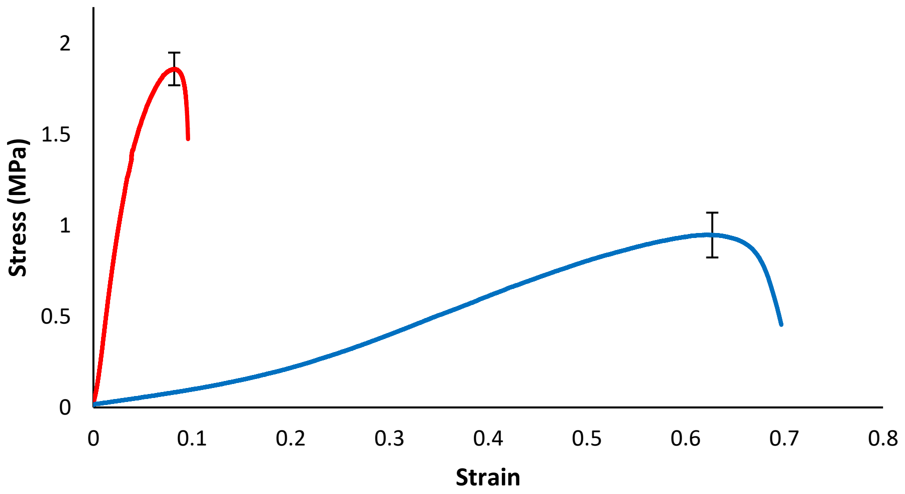

Figure S15; Stress strain curves (average of five samples) for **pEEA*15*/2** (1% wt) (red) and **pEEA*15*/2** (5% wt) (blue).

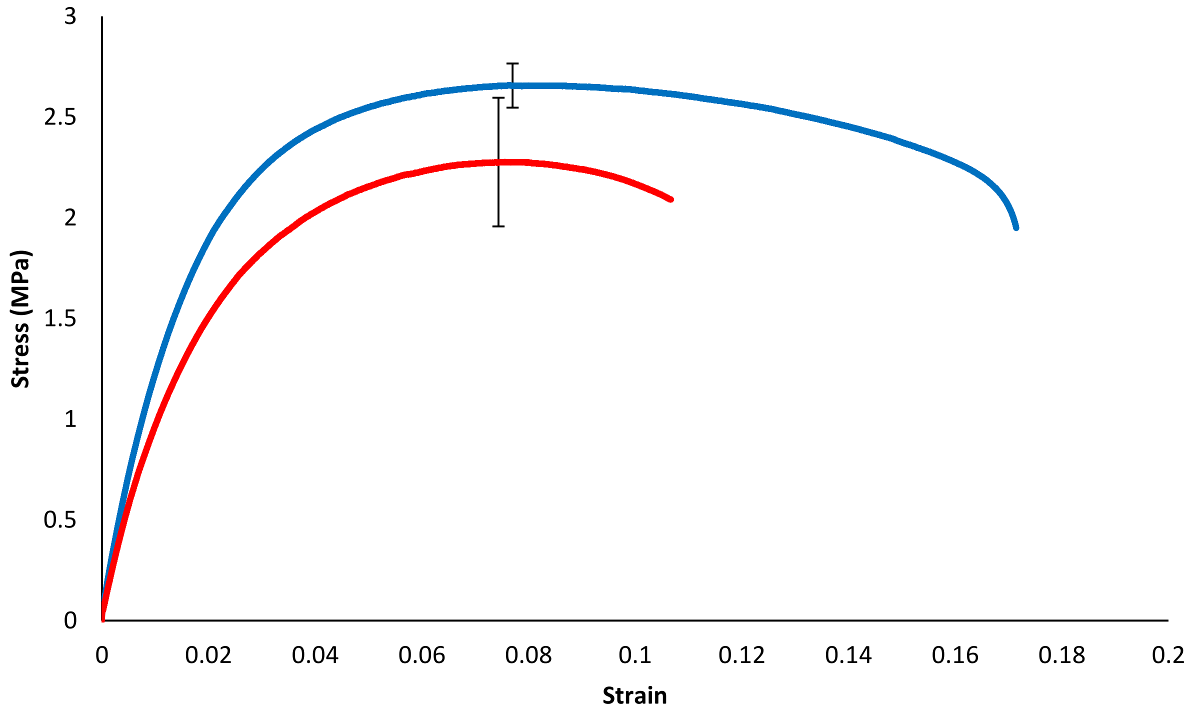

Figure S16; Stress strain curves (average of five samples) for **pEEA*15*/3** (1% wt) (red) and **pEEA*15*/3** (5% wt) (blue).

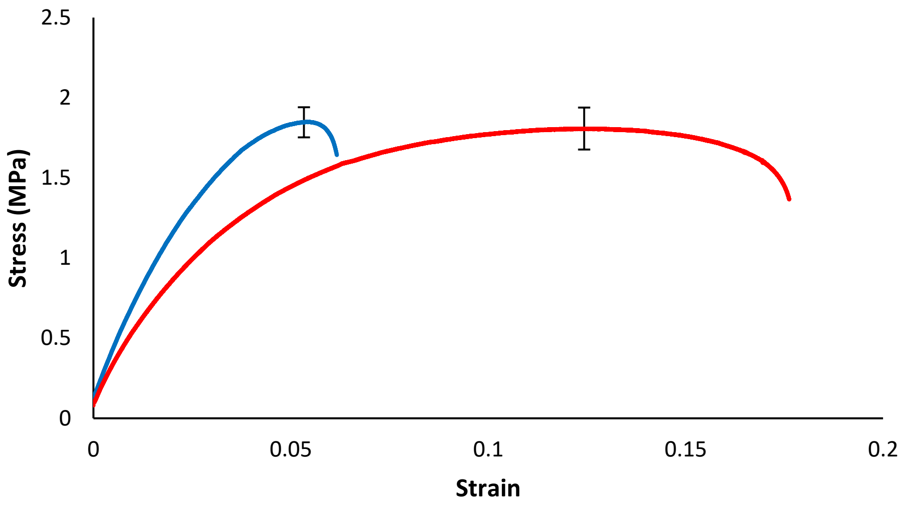

Figure S17; Stress strain curves (average of five samples) for **pEAA*15*** and **4** at 1% (red) and 5% (blue) wt.

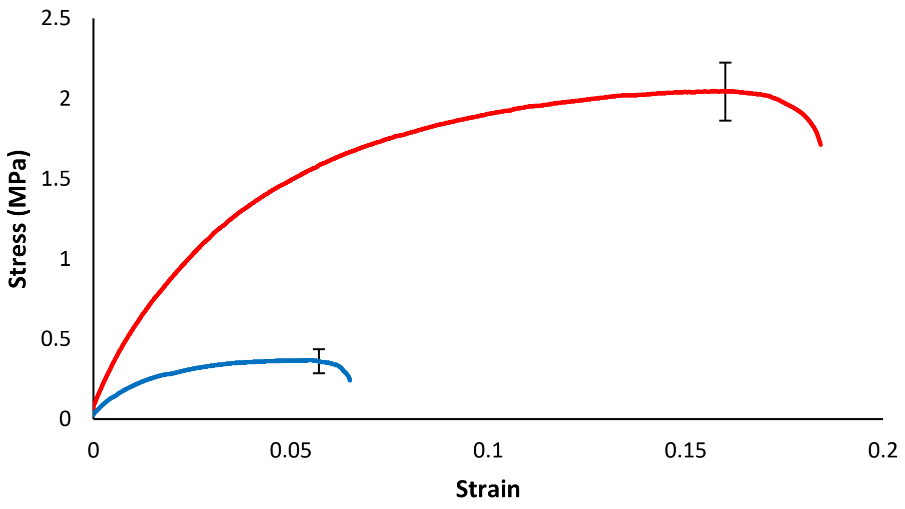

Figure S18; Stress strain curves (average of five samples) for **pEAA*15*** and **5** at 1% (red) and 5% (blue) wt.

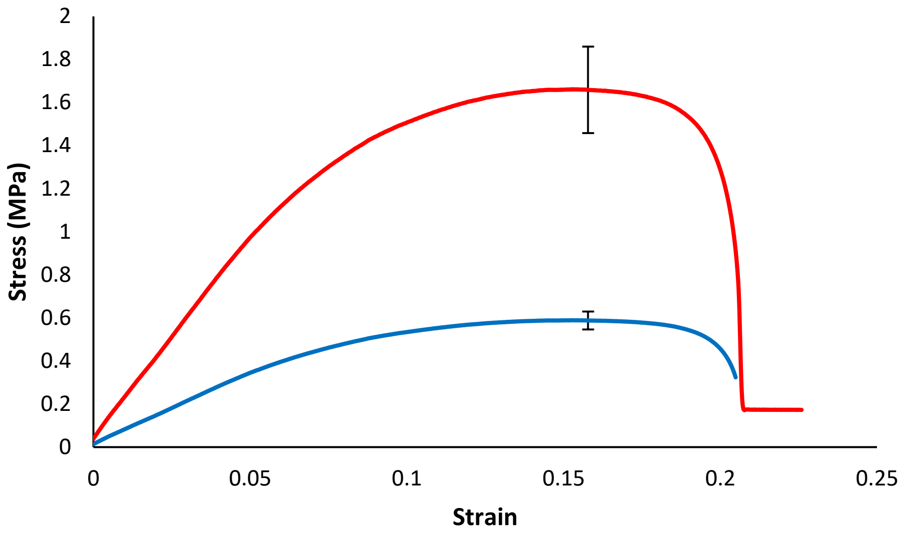

Figure S19; Stress strain curves (average of five samples) for **pEAA*15*** and **6** at 1% (red) and 5% (blue) wt.

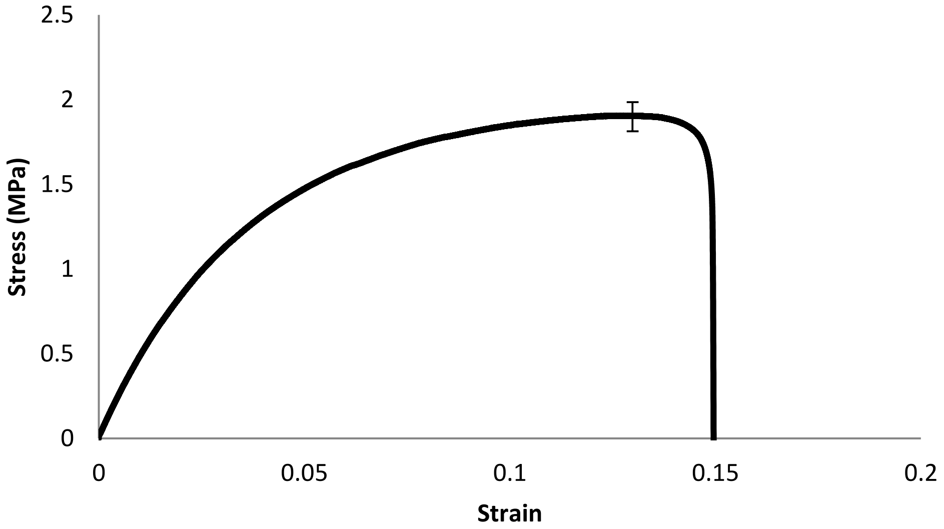

Figure S20; Stress strain curve (average of five samples) for **pEAA*15*** and **7** at 1% wt.

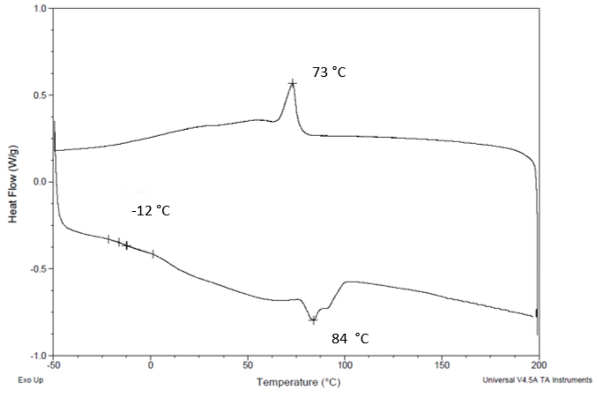

Figure S21; DSC heating scan (below) and subsequent cooling scan (above) of **pEEA*15***.

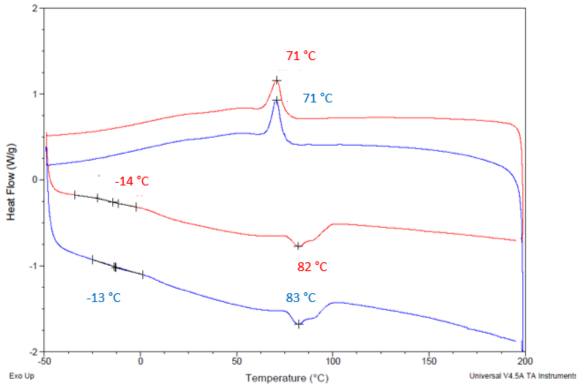

Figure S22; DSC heating/cooling scans of **pEEA*15*/1** at 1% (red) and 5% weight (blue).

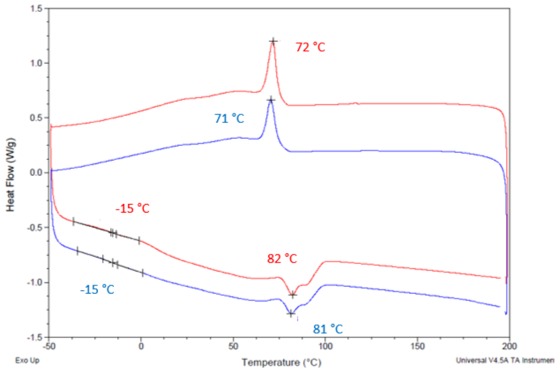

Figure S23; DSC heating/cooling scans of **pEEA*15*/2** at 1% (red) and 5% weight (blue).

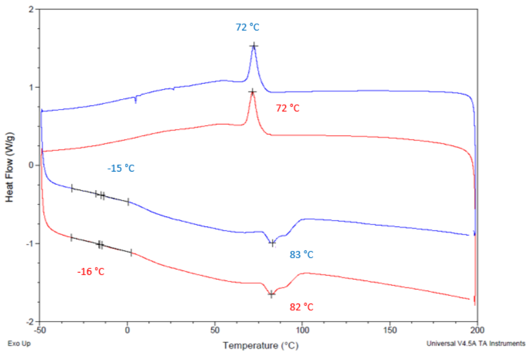

Figure S24; DSC heating/cooling scans of **pEEA*15*/3** at 1% (red) and 5% weight (blue).

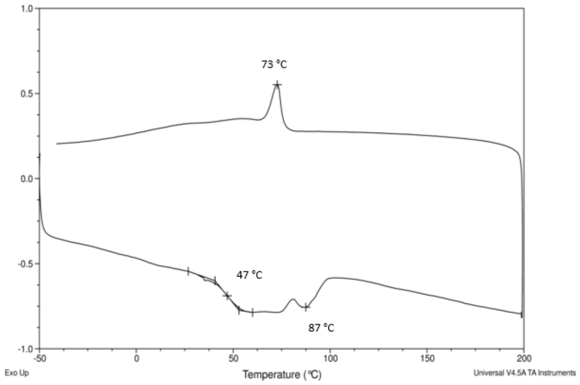

Figure S25; Relaxation DSC scan of **pEEA*15*** (48 hours after scan shown in **Figure S19**).

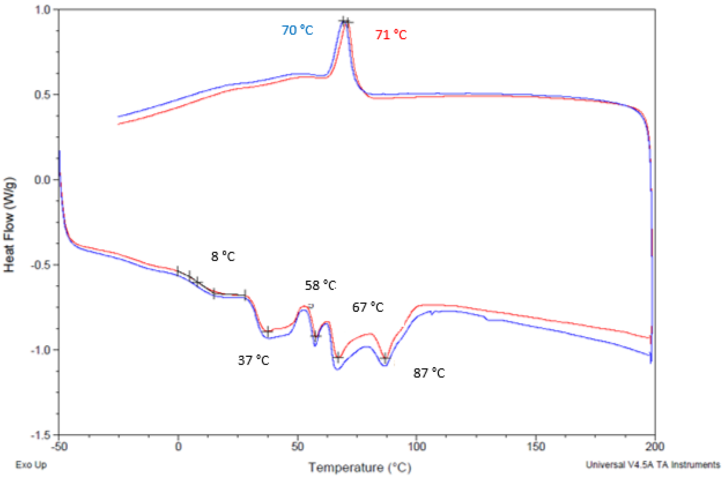

Figure S26; Relaxation DSC scans of **pEEA*15* /1** at 1% (red) and 5% wt. (blue) (taken 48 hours after scan shown in **Figure S20**).

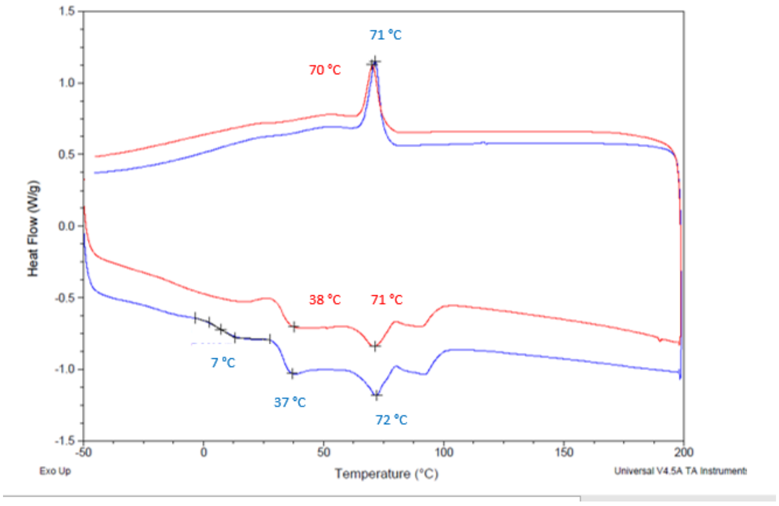

Figure S27; Relaxation DSC scan of **pEEA*15*/2** at 1% (red) and 5% wt. (blue) (taken 48 hours after scan shown in **Figure S21**).

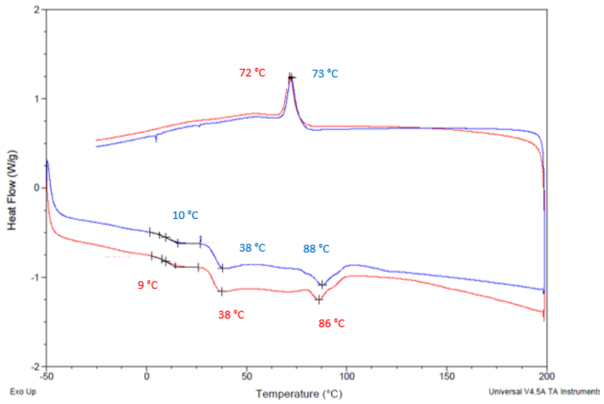

Figure S28; Relaxation DSC scan of **pEEA*15*/3** at 1% (red) and 5% wt. (blue) (taken 48 hours after scan shown in **Figure S22**).

Table S1; Tensile properties after heating fractured films at 50 °C for 8 hours.

Film System	% wt additive	Tensile Strength (MPa)	Fracture Stress (MPa)	Uniform Strain (%)	Strain to Fracture (%)	Energy absorbed (MPa)	Young's Modulus (MPa)
**pEEA*15***		0.12	0.12	1.56	1.60	0.002	5.68
**pEEA*15*/1**	5	0.09	0.09	0.53	0.56	0.001	13.12
**pEEA*15*/2**	1	0.57	0.57	1.50	1.58	0.013	23.23
**pEEA*15*/3**	5	0.88	0.88	2.30	2.42	0.013	18.61

Table S2; Tensile properties after heating fractured films at 60 °C for 2 hours.

Film System	% wt additive	Tensile Strength (MPa)	Fracture Stress (MPa)	Uniform Strain (%)	Strain to Fracture (%)	Energy absorbed (MPa)	Young's Modulus (MPa)
**pEEA*15***		0.39	0.37	1.67	1.74	0.008	14.22
**pEEA*15*/1**	5	-	-	-	-	-	-
**pEEA*15*/2**	1	0.72	0.63	4.43	5.26	0.03	17.97
**pEEA*15*/3**	5	1.12	1.12	2.29	2.37	0.021	28.53

Table S3; Tensile properties after pressing (0.98 MPa) fractured films for 8 hours.

Film System	% wt additive	Tensile Strength (MPa)	Fracture Stress (MPa)	Uniform Strain (%)	Strain to Fracture (%)	Energy absorbed (MPa)	Young's Modulus (MPa)
**pEEA*15***		1.00	0.84	6.15	7.38	0.06	26.31
**pEEA*15*/1**	5	1.35	0.93	6.75	9.68	0.10	21.83
**pEEA*15*/2**	1	0.20	0.15	1.07	1.23	0.007	18.60
**pEEA*15*/3**	5	1.95	0.96	5.62	7.48	0.14	41.43

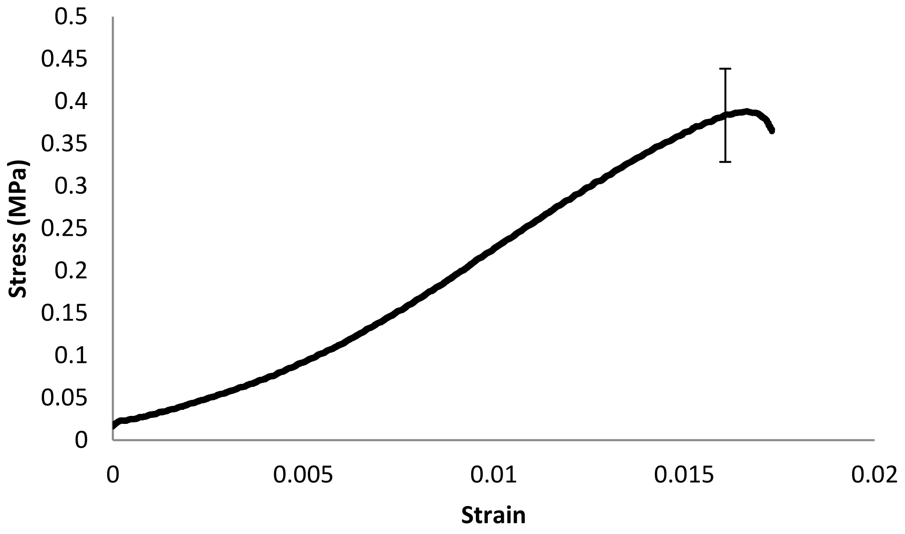

Figure S29; Stress-strain curve (average of five samples) for **pEAA*15*** after healing at 60 ˚C (2 hours)

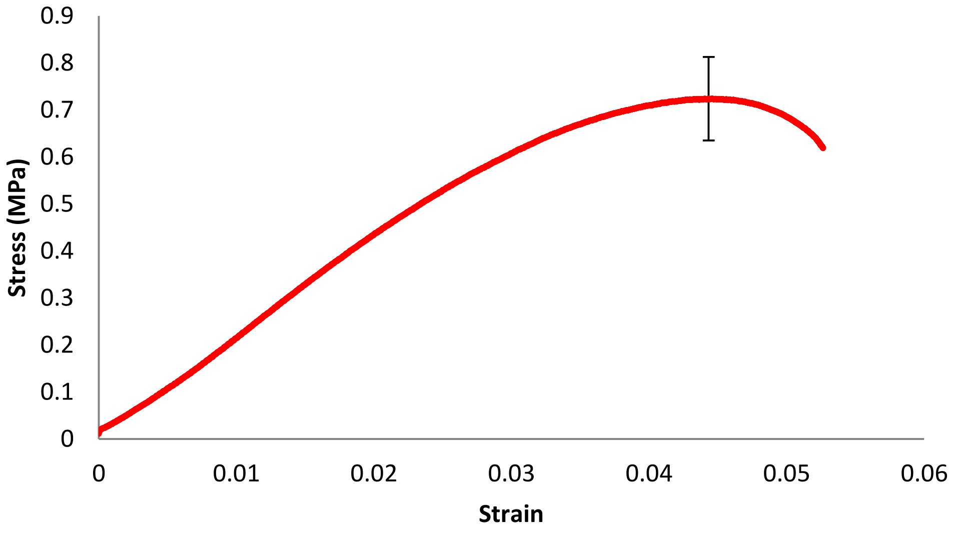

Figure S30; Stress-strain curve (average of five samples) for **pEAA*15*/2** (1% wt) after healing at 60 ˚C (2 hours)

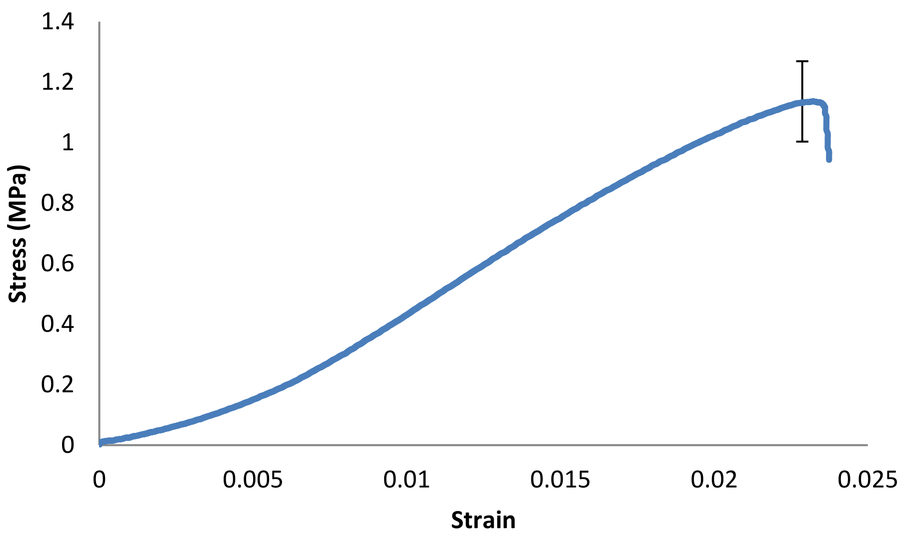

Figure S31; Stress-strain curve (average of five samples) for **pEAA*15*/3** (5% wt) after healing at 60 ˚C (2 hours)

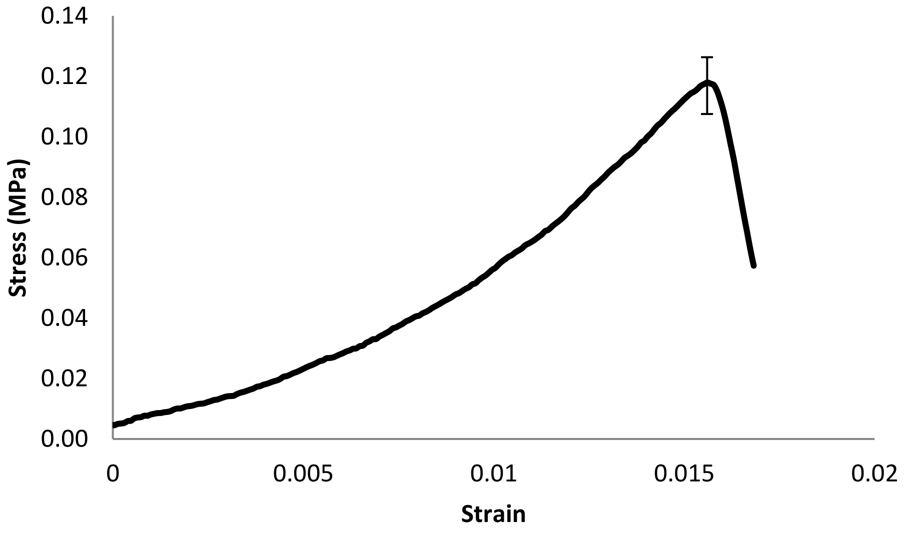

Figure S32; Stress-strain curve (average of five samples) for **pEAA*15*** after healing at 50 ˚C (8 hours).

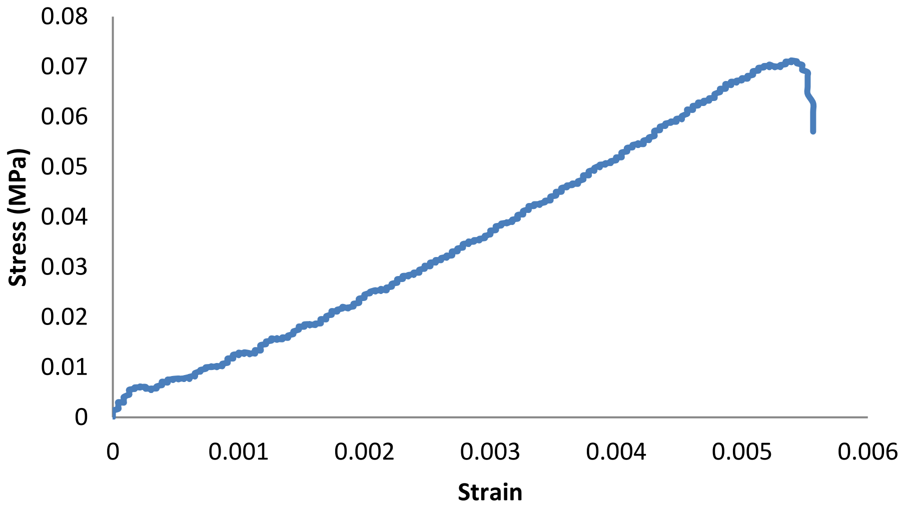

Figure S33; Stress-strain curve for **pEAA*15*/1** (5% wt) after healing at 50 ˚C (8 hours)

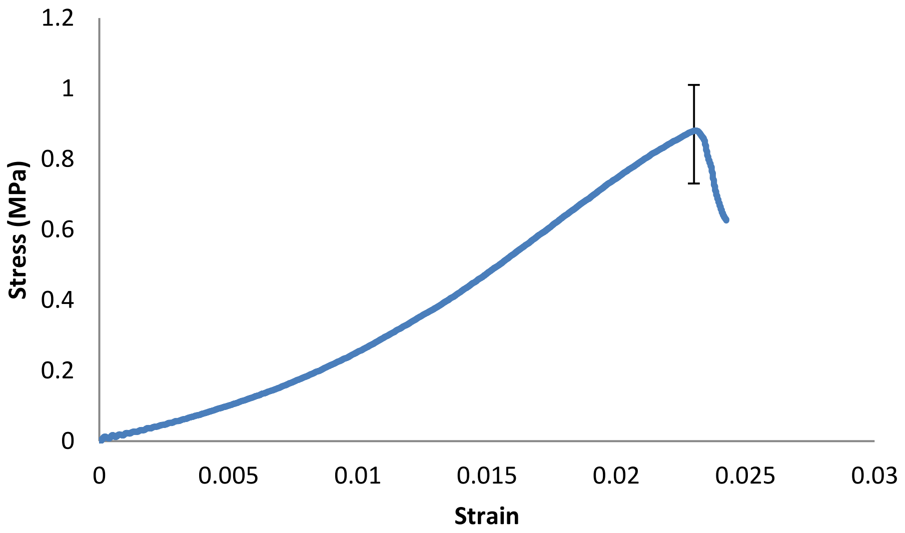

Figure S34; Stress-strain curve (average of five samples) for **pEAA*15*/3** (5% wt) after healing at 50 ˚C (8 hours)

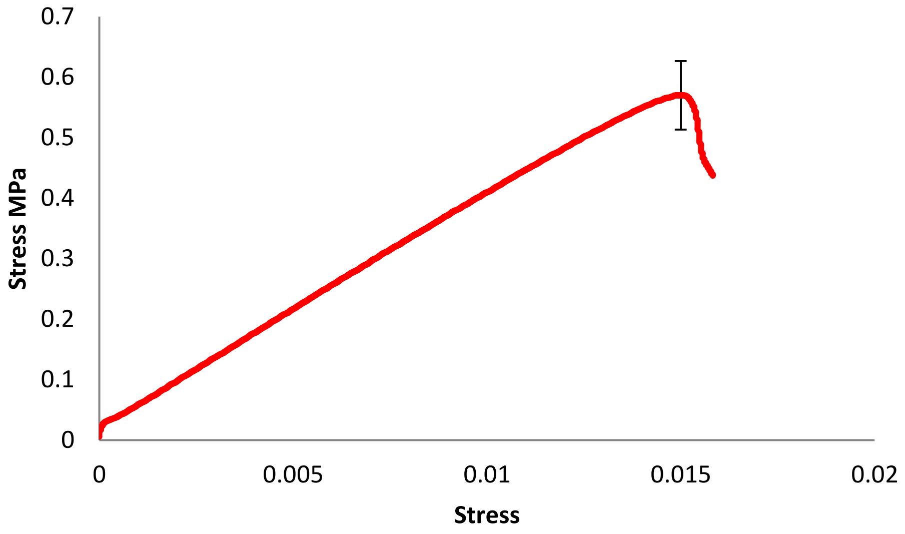

Figure S35; Stress-strain curve (average of five samples) for **pEAA*15*/2** (1% wt) after healing at 50 ˚C (8 hours)

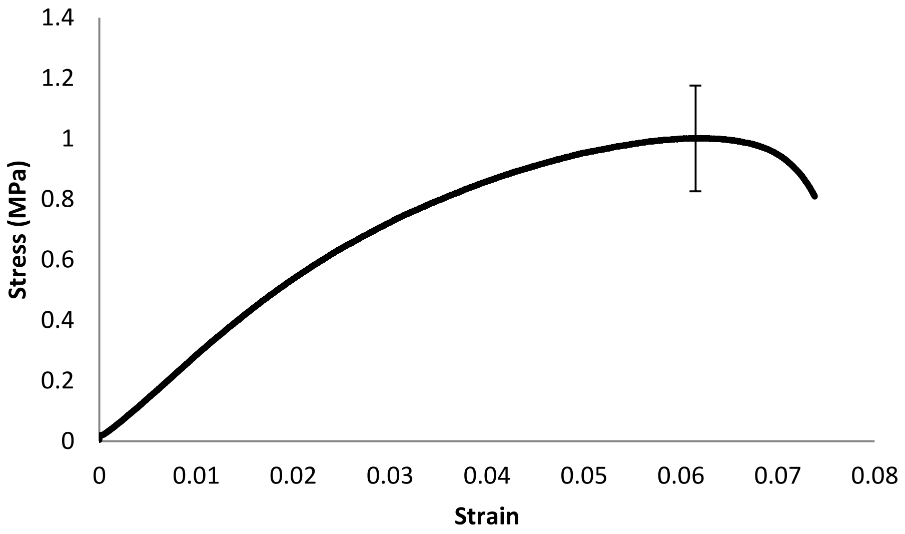

Figure S36; Stress-strain curve (average of five samples) for **pEAA*15*** after healing under pressure (0.98 MPa, 8 hours)

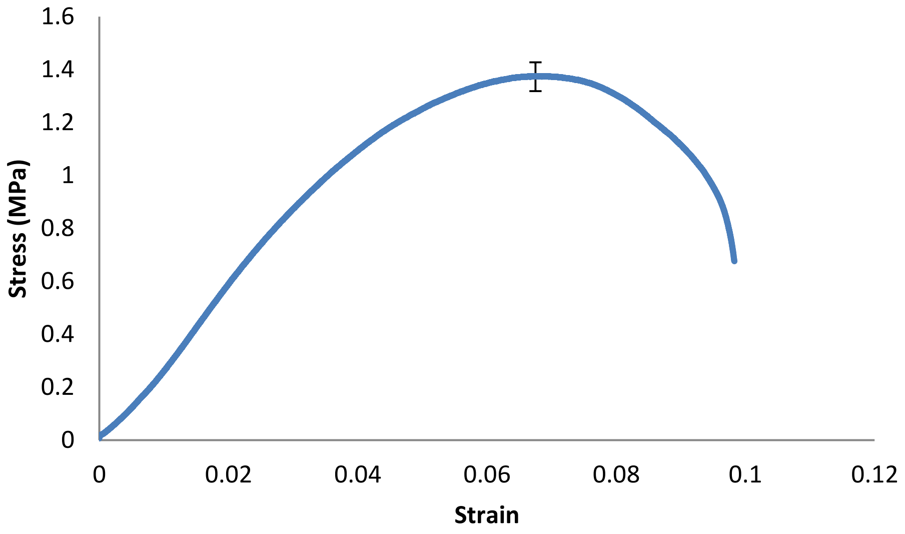

Figure S37; Stress-strain curve (average of five samples) for **pEAA*15*/1** (5% wt) after healing under pressure (0.98 MPa, 8 hours)

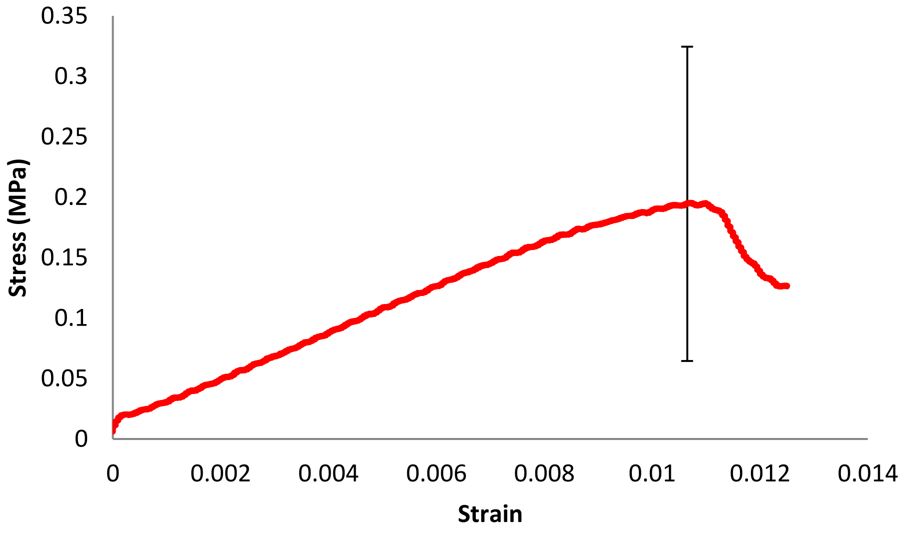

Figure S38; Stress-strain curve (average of five samples) for **pEAA*15*/2** (1% wt) after healing under pressure (0.98 MPa, 8 hours)

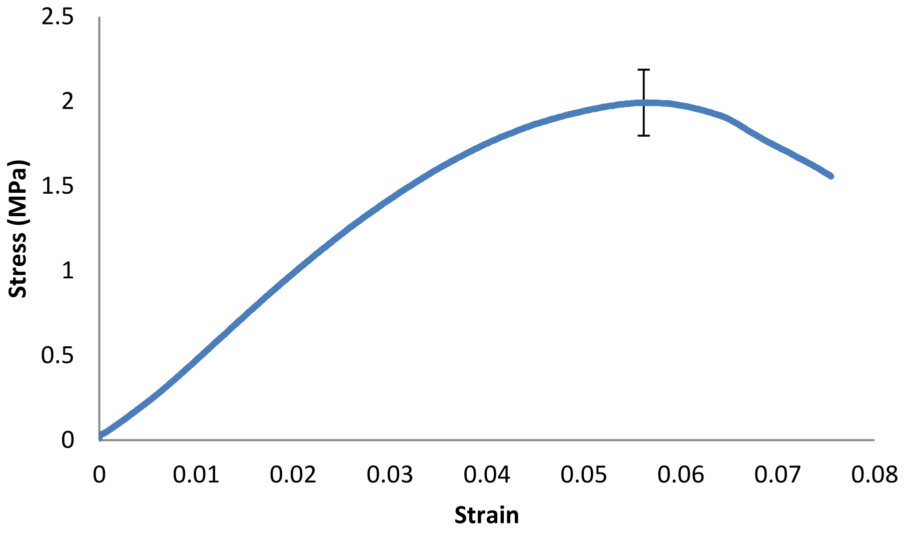

Figure S39; Stress-strain curve (average of five samples) for **pEAA*15/*3** (5% wt) after healing under pressure (0.98 MPa, 8 hours)

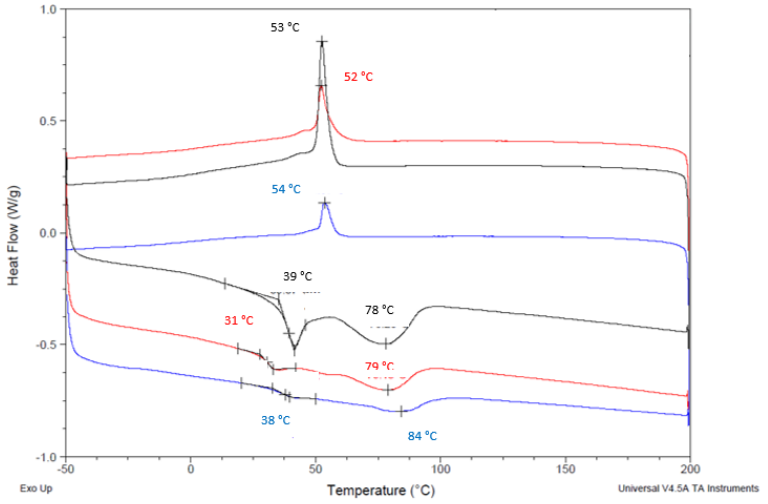

Figure S40; DSC thermograms for **pEAA*20*** (black) and **pEAA*20/*1** (blue) and **3** (red) each at 10% wt. The three lower traces are the heating scans and the three upper traces are the cooling scans.

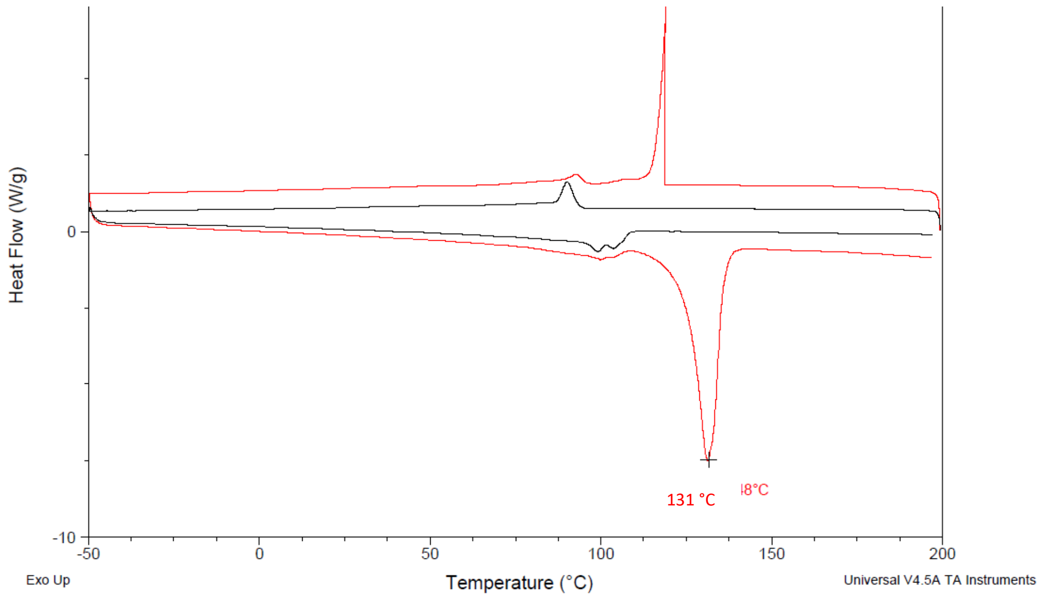

Figure S41; DSC thermograms for **pEAA5** (black) and **pEAA5/1** (red) at 2% wt.

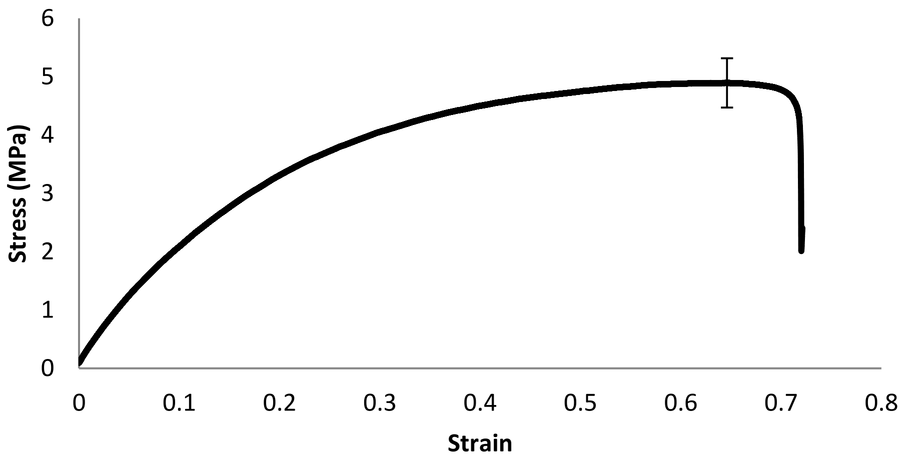

Figure S42; Stress-strain curve (average of five samples) of **pEAA*20***.

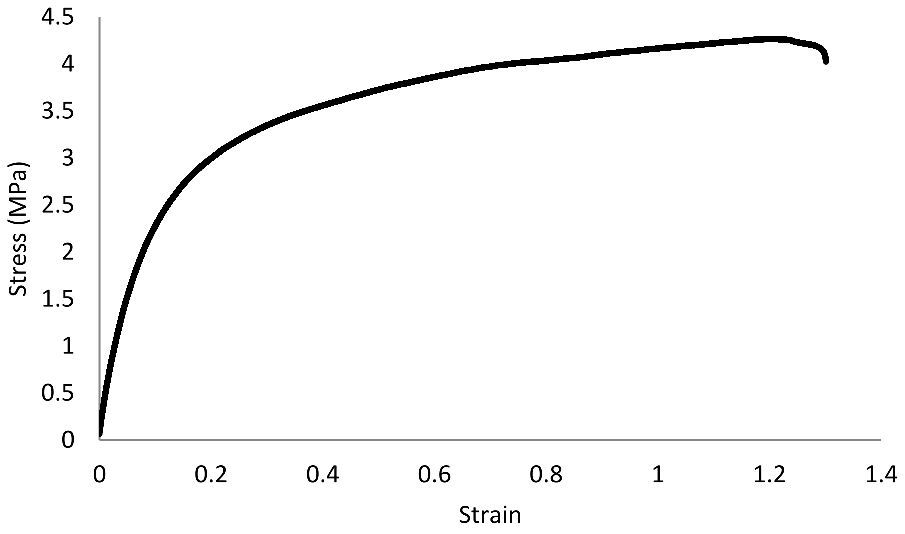

Figure S43; Stress-strain curve of **pEAA*20*/1** (10% wt).

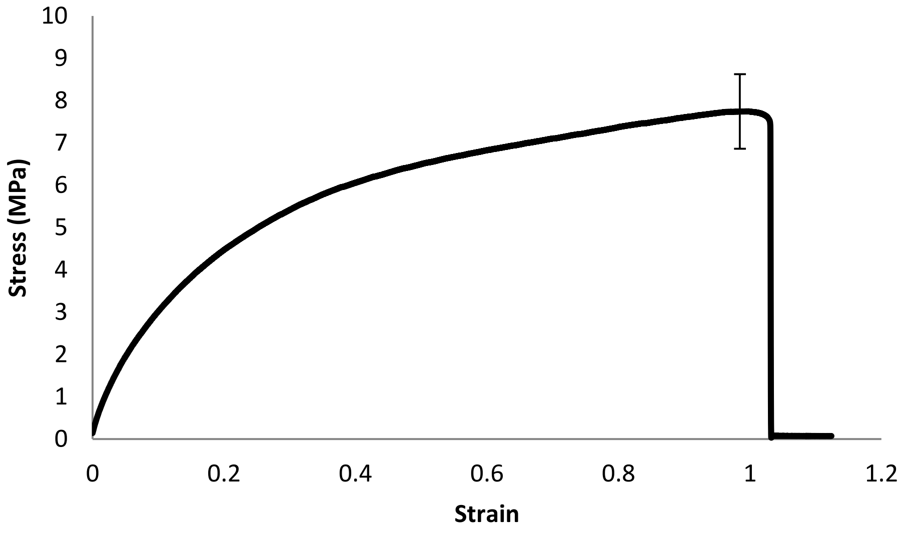

Figure S44; Stress-strain curve (average of five samples) of **pEAA*20*/3** (10% wt).

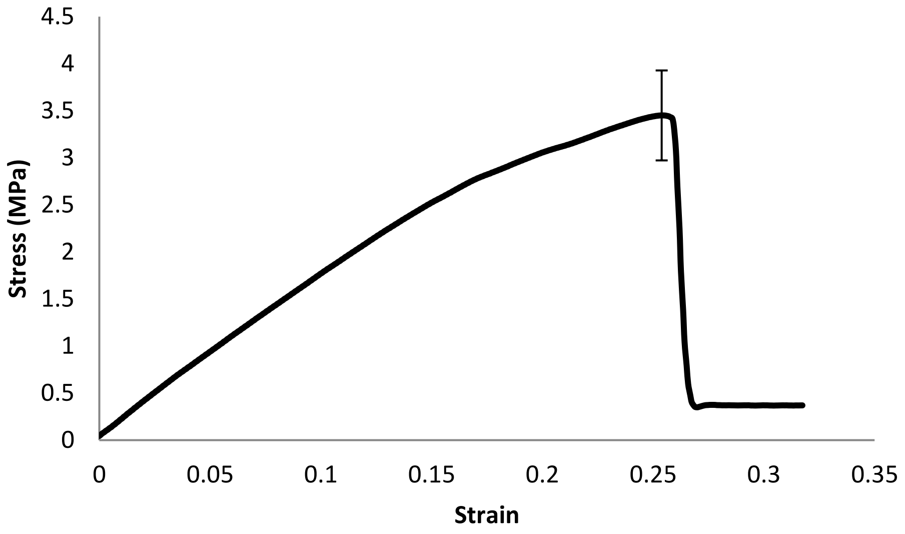

Figure S45; Stress-strain curve (average of five samples) of **pEAA*20*/3** (10% wt) after healing under pressure (0.98 MPa, 8 hours).
